# Selective and reversible 1,3-dipolar cycloaddition of 6-aryl-1,5-diazabicyclo[3.1.0]hexanes with 1,3-diphenylprop-2-en-1-ones under microwave irradiation

**DOI:** 10.3762/bjoc.16.218

**Published:** 2020-10-30

**Authors:** Alexander P Molchanov, Mariia M Efremova, Mariya A Kryukova, Mikhail A Kuznetsov

**Affiliations:** 1 Department of Organic Chemistry, Institute of Chemistry, Saint Petersburg State University, 7/9 Universitetskaya nab., St. Petersburg, 199034, Russia

**Keywords:** azomethine imines, cycloaddition, diazabicyclohexanes, diaziridines, regioselectivity

## Abstract

The first example of the cycloaddition of in situ-generated azomethine imine under microwave conditions is described. The reaction of 6-aryl-1,5-diazabicyclo[3.1.0]hexanes with 1,3-diphenylprop-2-en-1-ones proceeds regio- and stereoselectively giving mostly good yields of the corresponding perhydropyrazolopyrazoles. The products of the reaction undergo cycloreversion under the reaction conditions.

## Introduction

**Scheme 1:**
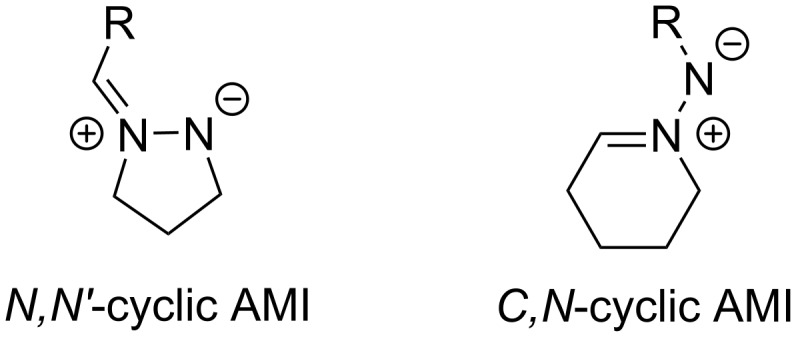
The two types of azomethine imines (AMI).

Cyclic azomethine imines (AMIs) are very useful synthetic blocks for the preparation of diverse dinitrogenated heterocycles by 1,3-dipolar cycloadditions under thermal or catalytic conditions [1–2]. In general, cyclic azomethine imines are classified into two types, depending on their structure: *N,N'*-cyclic AMIs with both nitrogen atoms included in the cyclic system and *C,N*-cyclic AMIs having the C=N motif in the cycle (Scheme 1) [3]. A wide range of pharmaceuticals, agrochemicals, and other biologically active compounds are prepared using different types of (3 + *n*) cycloadditions, mainly with alkenes and alkynes [3–7]. For example, *N,N'*-cyclic azomethine imines are precursors of biologically active bicyclic pyrazolidinone derivatives like LY186826 and its analogues with antibacterial activity [8–12].

The chemistry of azomethine imines has been actively investigated since the second half of the last century but their reactivity is still far less studied compared with nitrones or azomethine ylides – other allyl-anionic-type 1,3-dipoles [13–14]. Moreover, most researches focused on stable azomethine imines [3–4]. The most accessible and widely studied precursors for unstable azomethine imines are diaziridines, i.e., three-membered rings containing two nitrogen atoms [15]. Thus, the unstable *N,N'*-cyclic AMIs can be generated in situ by thermal opening of any C–N bond of the diaziridine ring in 6-aryl-substituted 1,5-diazabicyclo[3.1.0]hexanes or 7-aryl-substituted 1,6-diazabicyclo[4.1.0]heptanes, respectively [16–17]. For the thermal generation conditions, the 1,3-dipolar cycloadditions of AMIs were studied in detail only with the very active dipolarophiles: *N*-arylmaleimides, dialkyl fumarates, dicyanoethylene, diphenylcyclopropenone, aryl isocyanates, and aryl isothiocyanates [18–22]. Another approach to the generation of unstable AMIs from 6-aryl-1,5-diazabicyclo[3.1.0]hexanes is based on the catalytic cleavage of the C–N bond in the diaziridine ring under the influence of Lewis acids [23–24]. However, the selectivity of the cycloaddition for thermal or catalytic conditions can be different [25]. It was possible to obtain the cycloaddition products of unstable azomethine imines with carbon disulfide [26], diphenylketene [27], β-nitrostyrenes [28–29], if ionic liquids (IL) were used as reaction media. The reaction of β-nitrostyrenes with azomethine imines proceeds giving mixtures of Michael and *anti*-Michael-type adducts, containing a nitro group at the C^2^- or C^1^-position of the hexahydropyrazolo[1,2-*a*]pyrazole ring, respectively, apparently through zwitterionic intermediates [28–29]. At the same time, the cycloaddition of azomethine imine to 1,3-diphenylprop-2-en-1-ones (chalcones) in IL proceeds stereo- and regioselectively giving two diastereomeric bicyclic *anti*-Michael-type cycloadducts [28]. Very recently, a catalytic enantioselective (3 + 2) cycloaddition of diazabicyclohexanes with chalcones was carried out [30]. 1,5-Diazabicyclo[3.1.0]hexanes have been employed as precursors for 1,3-dipoles not only in (3 + 2) but also in (3 + 3) cycloadditions. It was shown that the Lewis acid-catalyzed reaction of diaziridines with donor–acceptor cyclopropanes and aziridines affords the perhydropyridazine or triazine derivatives, respectively, in good yields [31–33].

The use of microwave irradiation in organic synthesis complies with the principles of green chemistry and has attracted much interest. For 1,3-dipolar cycloaddition reactions microwave irradiation not only allows to reduce the reaction time and to increase yields, but in some cases also can affect the selectivity of the reaction [34–36]. The efficiency of microwave irradiation has been shown for transformations of azomethine imines [37–39]. However, only the conversion to pyrazolines and the dimerization were investigated previously for diaziridines under microwave conditions [40–41]. The cycloaddition reactions of unstable azomethine imines generated from diaziridines were investigated using only convection heating.

The aim of the current work is to investigate the regio- and diastereoselectivity of the microwave-assisted (3 + 2) cycloaddition of 6-aryldiazabicyclo[3.1.0]hexanes with 1,3-diphenylprop-2-en-1-ones.

## Results and Discussion

**Scheme 2:**
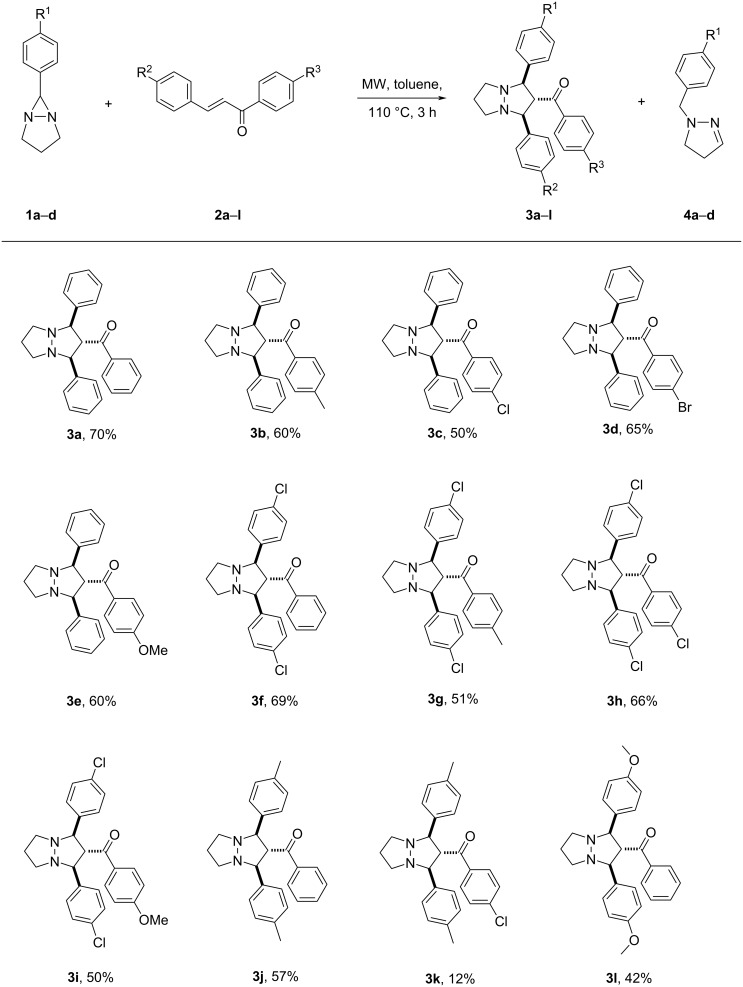
Reaction of 1,5-diazabicyclo[3.1.0]hexanes **1a–d** with diarylpropenones **2a–l**.

**Figure 1:**
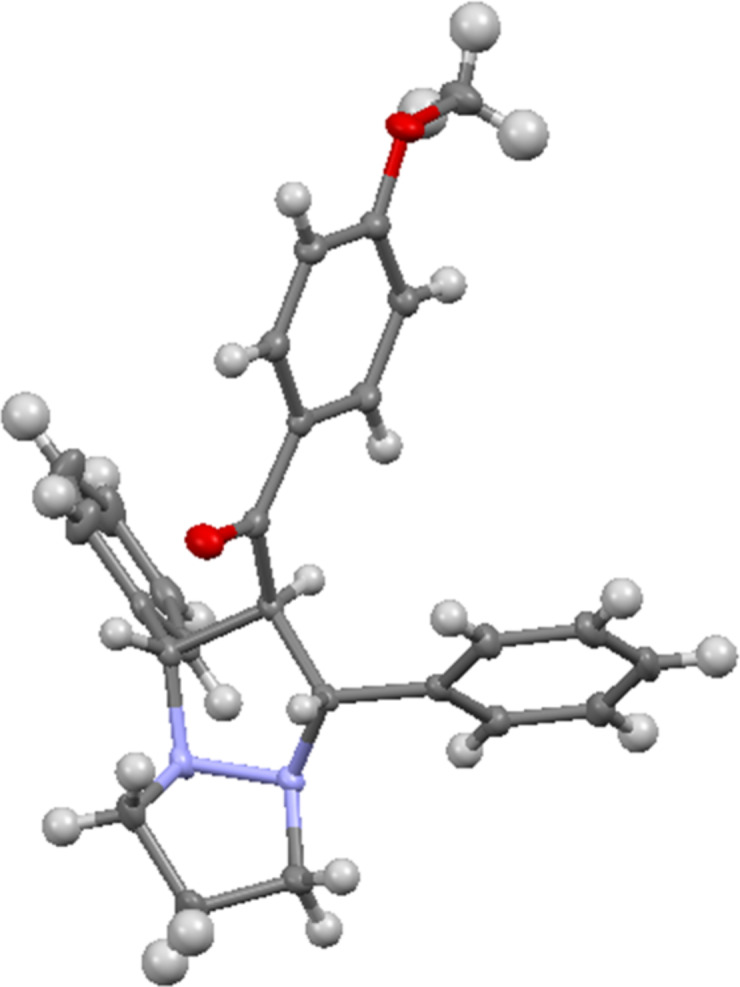
Single-crystal X-ray structure of compound **3e**.

**Figure 2:**
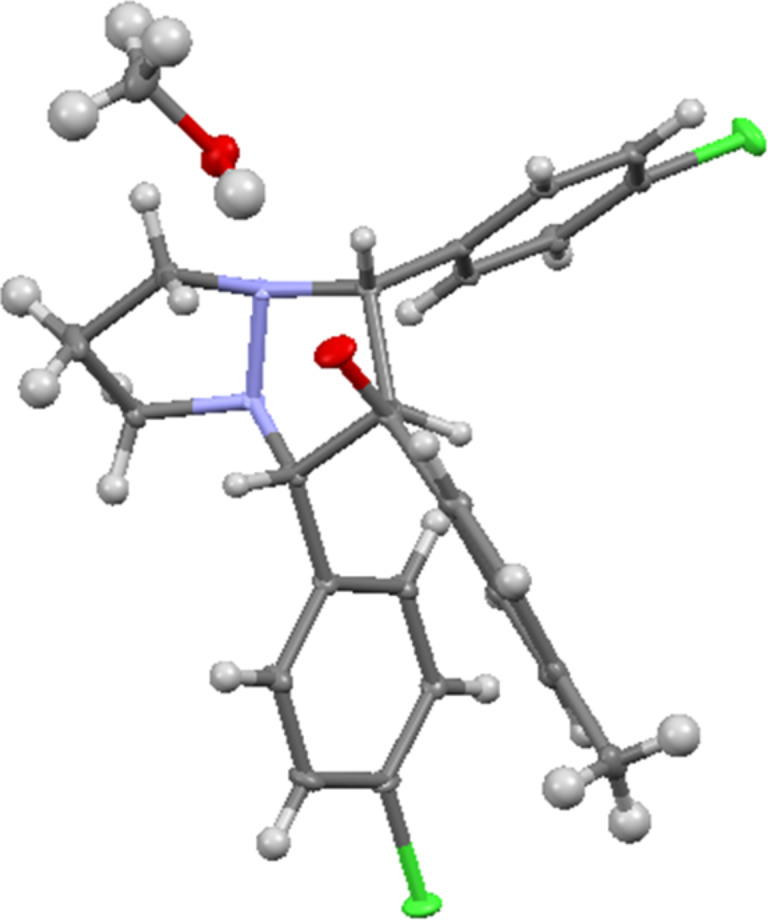
Single-crystal X-ray structure of compound **3g**.

Herein, we report the 1,3-dipolar cycloaddition reaction of 6-aryl-1,5-diazabicyclo[3.1.0]hexanes **1a–d** (DABCH) with 1,3-diarylpropenones **2a–l** at 110 °С under microwave irradiation using a microwave oven Discover SP in a 10 mL glass reactor. It was found that under these conditions the (3 + 2) cycloaddition products – perhydropyrazolopyrazoles **3a–l** are formed as single diastereomers mostly in good yields (up to 70%). In some cases, the pyrazolines **4a–d** are obtained in small quantities (Scheme 2). Pyrazolines are typical byproducts observed in transformations of 6-aryldiazabicyclo[3.1.0]hexanes and are formed during the isomerization of unstable intermediate azomethine imines, generated under the reaction conditions [16,20,40]. The reaction products were separated by column chromatography and their structures proved by spectral data. Because there are contradictory data on the product structures of the reaction of DABCH with chalcones in the literature [11,13], we have carried out the careful structure analysis of the obtained compounds. So, in the ^1^H NMR spectrum of compound **3a** there are only two signals for the substituted pyrazolidine ring – a doublet (2H) at 4.48 ppm (*J* = 7.4 Hz) and a triplet (1H) at 4.39 ppm (*J* = 7.4 Hz). Accordingly, two characteristic signals for carbon atoms of the pyrazolidine ring are present in the ^13^C NMR spectrum – at 72.2 (2СН) and 68.7 (СН) ppm. The spatial configuration of the adducts **3e** and **3g** was further determined by single crystal X-ray diffraction analysis (Figure 1 and Figure 2). All products **3a–l** had similar NMR spectra and they are assumed to have the same relative configuration. It is of note, that the *trans*-configuration of the substituents in the starting chalcones completely remains in the cycloadducts, which is a characteristic feature of concerted processes.

It has been found earlier that the electronic properties of the substituted aryl group of diaziridines and chalcones showed a profound influence on the yields of a catalytic reaction (scandium triflate with a chiral ligand); when a phenyl or *p*-chloro/methylphenyl-substituted diaziridine was employed, the cycloaddition products were obtained in only 11–24% yields, and good yields were achieved mostly with diaziridines containing electron-donating substituents [30]. In our case the products were obtained in good yields in all cases except for compound **3k** (12%), that was mainly due to difficulties in the separation of the product from a substantial quantity of formed pyrazoline **4c**.

**Scheme 3:**
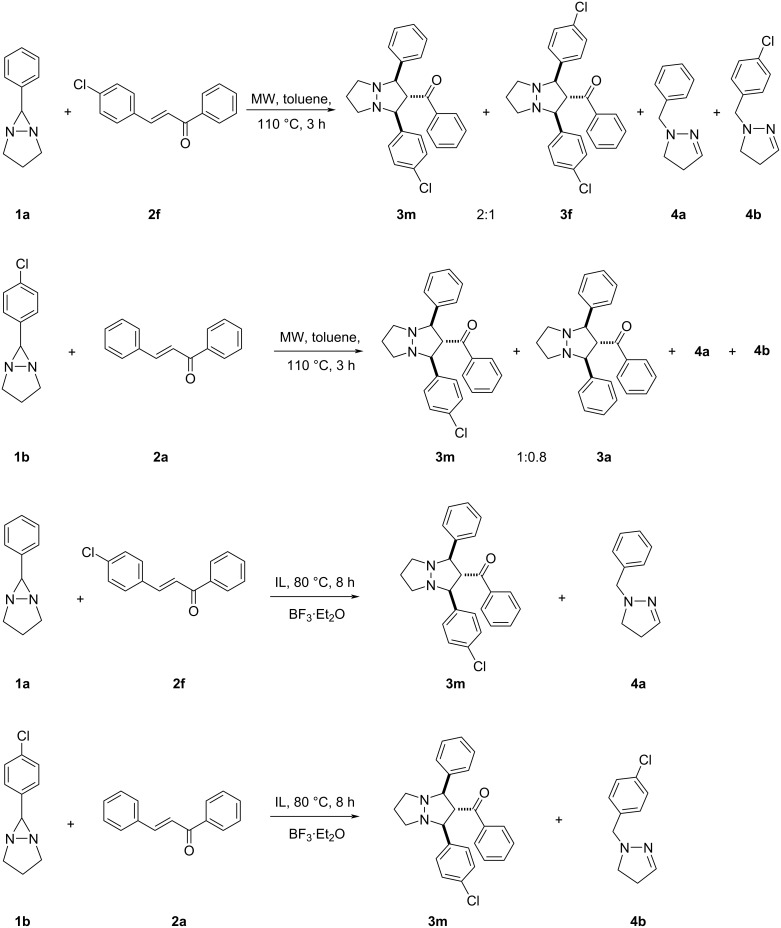
Control experiments.

It is remarkable that the reaction of 6-phenyl-1,5-diazabicyclo[3.1.0]hexane (**1a**) with diarylpropenone **2f** under the same conditions (microwave irradiation, 110 °С, toluene, 3 h) affords the two adducts **3f** and **3m** in a 1:2 ratio and the two pyrazolines **4a** and **4b**. At the same time, the reaction of DABCH **1b** with chalcone **2a** also gives adducts **3m** and **3a** and the same pyrazolines **4a** and **4b**. As it was marked in the literature, the reactions were expected to occur in ionic liquids more selectively than in organic solvents owing to the stabilization of polar species in the ionic medium. Therefore, we carried out the reaction of DABCH **1a** with propenone **2f** in bmimCl in the presence of BF_3_·Et_2_O at 80 °C for 8 h, that led to the formation of adduct **3m** and pyrazoline **4a**. The reaction of DABCH **1b** and propenone **2a** under the same conditions resulted in the formation of adduct **3m** and pyrazoline **4b** (Scheme 3).

**Scheme 4:**
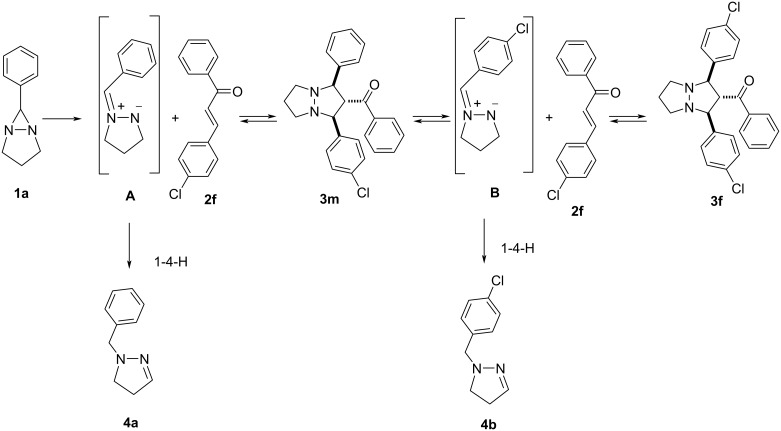
Mechanistic hypothesis for cycloaddition and cycloreversion reactions of diazabicyclohexane **1a** with propenone **2f**.

Thus, the azomethine imine **A**, formed by heating of DABCH **1a**, either reacts with propenone **2f** giving the adduct **3m** or undergoes isomerization affording pyrazoline **4a**. On the basis of this observation we suppose that the reaction products undergo cycloreversion, i.e., under the reaction conditions the adduct **3m** may undergo a ring-opening with the liberation of the starting propenone **2f** and the new isomeric azomethine imine **B**. The latter is able to isomerize into pyrazoline **4b** or it reacts with propenone **2f** leading to adduct **3f** (Scheme 4).

**Scheme 5:**
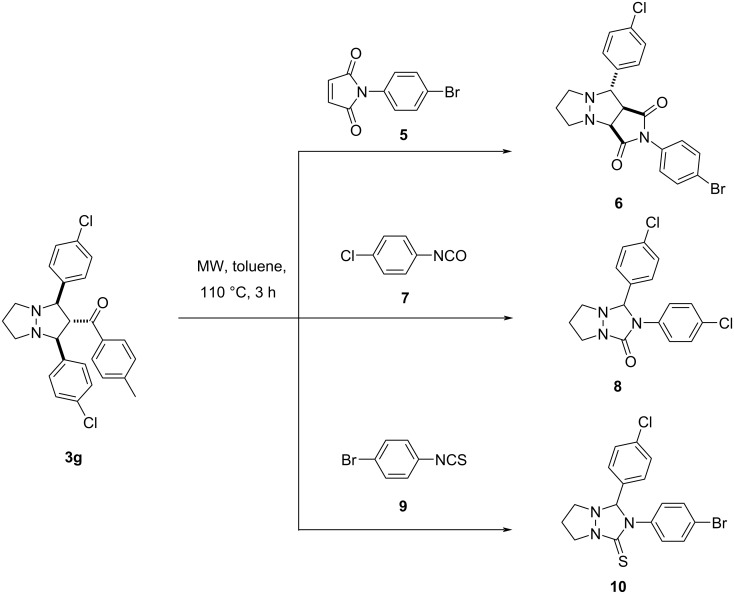
Experiments on the trapping of azomethine imine, generated from pyrazolopyrazole **3g**.

To prove this assumption, we decided to study alternative methods for the generation of azomethine imines from adducts **3**. For this purpose, pyrazolopyrazole **3g** was heated in toluene at 110 °C for 3 hours under microwave irradiation in the presence of dipolarophiles such as maleimide **5**, isocyanate **7**, and isothiocyanate **9** (Scheme 5). The corresponding adducts **6**, **8**, and **10** were obtained in good yields in all cases. The cycloreversion of adducts formed from azomethine imines, generated of 6-aryl-1,5-diazabicyclo[3.1.0]hexanes has been earlier assumed for the reaction of AMI with isatine, where instead of the expected fused heterocycles, a mixture of substituted pyrazolines and corresponding pyrazoles was obtained [42], and also for the reaction with arylmethylidenemalononitriles [43]. It was assumed that the expected compounds initially had formed, but under the reaction conditions, they underwent a ring opening with the liberation of the aromatic aldehyde and formation of the new azomethine imines.

## Conclusion

The cycloaddition of azomethine imines, generated from 6-aryl-1,5-diazabicyclo[3.1.0]hexanes under microwave heating, with 1,3-diarylpropenones occurs regioselective with the formation of 2-benzoyl-substituted pyrazolo[1,2-*a*]pyrazoles (Michael-type adducts). For the first time it was shown, that under heating the cycloadducts can undergo cycloreversion with the generation of new azomethine imines, which can be trapped with suitable dipolarophiles.

## Supporting Information

File 1 Experimental and characterization data of all new compounds.
