# Supplementary material for: Selective and reversible 1,3-dipolar cycloaddition of 6-aryl-1,5-diazabicyclo[3.1.0]hexanes with 1,3-diphenylprop-2-en-1-ones under microwave irradiation
Source: Beilstein J Org Chem. 2020 Oct 30;16:2679–86. doi: 10.3762/bjoc.16.218 (PMC7607433; doi:10.3762/bjoc.16.218)
Supplement: File 1 — Experimental and characterization data of all new compounds. [file Beilstein_J_Org_Chem-16-2679-s001.pdf]

BEILSTEIN JOURNAL OF ORGANIC CHEMISTRY

Supporting Information

for

Selective and reversible 1,3-dipolar cycloaddition of 6-aryl-1,5-diazabicyclo[3.1.0]hexanes with 1,3-diphenylprop-2-en-1-ones under microwave irradiation

Alexander P. Molchanov, Mariia M. Efremova, Mariya A. Kryukova
and Mikhail A. Kuznetsov

Beilstein J. Org. Chem. **2020,** *16,* 2679–2686. doi:10.3762/bjoc.16.218

Experimental and characterization data of all new compounds

License and Terms: This is a supporting information file under the terms of the Creative Commons Attribution License (https://creativecommons.org/licenses/by/4.0). Please note that the reuse, redistribution and reproduction in particular requires that the authors and source are credited.
The license is subject to the *Beilstein Journal of Organic Chemistry* terms and conditions: (https://www.beilstein-journals.org/bjoc)

General remarks

All reactions were performed in anhydrous solvents under an argon atmosphere. Reaction progress was monitored using thin layer chromatography (TLC) on precoated Silufol UV–254 plates. The IR spectra were measured on a Shimadzu FT-IR spectrometer IRAffinity-1. ^{1}H and ^{13}C NMR spectra were recorded in $CDCl_3$ or DMSO-d_6 using a Bruker Avance 400 spectrometer. HRMS spectra were obtained with a Bruker-maXis (QTOF). An Xcalibur Eos diffractometer was used for X-ray analysis. All syntheses under microwave irradiation were performed in a microwave oven Discover SP in a 10 mL glass reactor. Diazabicyclohexanes were prepared using known procedures [1-3]. The characteristics of the previously described compounds **3a**, **3m**, and **6** correspond to the data described in the literature [4,5].

No	DABCH	chalcone	R^1	R^2	R^3	**2/1**	Product, **3**	**3/4**^{a}	Yield **3** ^{b}, %
1	**1a**	**2a**	H	H	H	1.3	**3a**	4/1	70
2	**1a**	**2b**	H	H	Me	1.5	**3b**	3/1	60
3	**1a**	**2c**	H	H	Cl	2.0	**3c**	-	50
4	**1a**	**2d**	H	H	Br	1.3	**3d**	-	65
5	**1a**	**2e**	H	H	OMe	2.0	**3e**	5/1	60
6	**1b**	**2f**	Cl	Cl	H	0.9	**3f**	2/1	69
7	**1b**	**2g**	Cl	Cl	Me	1.0	**3g**	3/1	51
8	**1b**	**2h**	Cl	Cl	Cl	1.5	**3h**	-	66
9	**1b**	**2i**	Cl	Cl	OMe	1.3	**3i**	1/1	50
10	**1c**	**2j**	Me	Me	H	1.5	**3j**	1.5/1	57
11	**1c**	**2k**	Me	Me	Cl	1.2	**3k**	1/7	12
12	**1d**	**2l**	OMe	OMe	H	2.1	**3l**	2/1	42

^{a} -From ^{1}H NMR spectra of the crude reaction mixture; ^{b} – isolated yield.

General procedure for the reaction of diazabicyclohexanes and diarylpropenones. A mixture 1 mmol of the diazabicyclohexane **1a–d** and *n* mmol (*n* as defined in the table above) of the propenone **2a–l** in 4 mL of toluene was heated at 110 °C under microwave irradiation during 3 hours. Then, toluene was removed under reduced pressure and the products were obtained by column chromatography on silica gel using gradient elution with hexane/ethyl acetate.

((1*RS*,3*SR*)-1,3-Diphenyltetrahydro-1*H*,5*H*-pyrazolo[1,2-*a*]pyrazol-2-yl)(phenyl)methanone (3a), yield 260 mg (70%), light yellow solid, m.p. 86-87 °C. R_f 0.50 (hexane:ethyl acetate 4:1). IR: ν = 2980, 2853, 1676 s, 1598 s, 1488, 1453, 1370, 1323, 1283, 1252, 1122, 1102, 767, 749, 699 cm^{-1}; ^{1}H NMR (400 MHz, $CDCl_3$): δ 7.52-7.40 (m, 7H, Ar), 7.35-7.20 (m, 8H, Ar), 4.48 (d, 2H, *J* 7.4 Hz, CH), 4.39 (t, *J* 7.4 Hz, 1H, CH), 3.39-3.30 (m, 2H, CH_2), 3.26-3.18 (m, 2H, CH_2),

2.42-2.31 (m, 1H, CH_2), 2.16-2.04 (m, 1H, CH_2). ^{13}C NMR (100 MHz, $CDCl_3$): δ 199.8 (CO), 147.1 (C), 136.6 (C), 133.4 (C), 128.8 (3CH), 128.7 (4CH), 128.3 (2CH), 127.6 (2CH), 127.4 (4CH), 72.2 (2CH), 68.7 (CH), 51.1 (2CH_2), 23.9 (CH_2).

HRMS (ESI): *m/z* calcd. for $C_{25}H_{25}N_2O$ $(M+H)^+$ 369.1967, found 369.1968.

The characteristics correspond to described in the literature [4].

((1*RS*,3*SR*)-1,3- Diphenyltetrahydro-1*H*,5*H*-pyrazolo[1,2-*a*]pyrazol-2-yl)(*p*-tolyl)methanone (3b), yield 230 mg (60%), light yellow solid, m.p. 93-94 °C. R_f 0.60 (hexane:ethyl acetate 3:1). IR: ν = 2992, 2923, 2862, 1659 s, 1601 s, 1488, 1452, 1410, 1377, 1348, 1324, 1299, 1287, 1183 s, 1105, 1011, 746, 699 s cm^{-1}. ^{1}H NMR (400 MHz, DMSO-d_6): δ 7.41-7.36 (m, 4H, Ar), 7.33-7.20 (m, 8H, Ar), 7.10-7.05 (m, 2H, Ar), 4.37 (d, 2H, *J* 7.3 Hz, CH), 4.24 (t, *J* 7.3 Hz, 1H, CH), 3.11-3.00 (m, 4H, CH_2), 2.35-2.23 (m, 1H, CH_2), 2.25 (s, 3H, CH_3), 1.95-1.85 (m, 1H, CH_2). ^{13}C NMR (100 MHz, DMSO-d_6): δ 199.2 (CO), 144.7 (C), 142.7 (2C), 134.3 (C), 129.6 (2CH), 128.9 (4CH), 128.8 (4CH), 127.7 (4CH), 71.9 (2CH), 68.7 (CH), 50.8 (2CH_2), 23.9 (CH_2), 21.5 (CH_3).

HRMS (ESI): *m/z* calcd. for $C_{26}H_{27}N_2O$ $(M+H)^+$ 383.2118, found 383.2110.

(4-Chlorophenyl)((1*RS*,3*SR*)-1,3-diphenyltetrahydro-1*H*,5*H*-pyrazolo[1,2-*a*]pyrazol-2-yl)methanone (3c), yield 200 mg (50%), yellow solid, m.p. 100-101 °C. R_f 0.48 (hexane:ethyl acetate 3:1). IR: ν = 2989, 2884, 1662 s, 1584 s, 1488, 1452, 1401, 1346, 1286, 1206, 1180, 1093 s, 1008 s, 861, 815, 748, 699 s cm^{-1}. ^{1}H NMR (400 MHz, $CDCl_3$): δ 7.50-7.42 (m, 4H, Ar), 7.38-7.32 (m, 2H, Ar), 7.30-7.25 (m, 6H, Ar), 7.24-7.12 (m, 2H, Ar), 4.44 (d, 2H, *J* 7.4 Hz, CH), 4.29 (t, *J* 7.4 Hz, 1H, CH), 3.34-3.26 (m, 2H, CH_2), 3.23-3.14 (m, 2H, CH_2), 2.38-2.30 (m, 1H, CH_2), 2.12-2.03 (m, 1H, CH_2). ^{13}C NMR (100 MHz, $CDCl_3$): δ 198.8 (CO), 142.0 (2C), 140.0 (C), 134.9 (C), 130.2 (2CH), 128.7 (4CH), 128.6 (2CH), 127.6 (2CH), 127.4 (4CH), 72.0 (2CH), 69.2 (CH), 51.0 (2CH_2), 23.8 (CH_2).

HRMS (ESI): *m/z* calcd. for $C_{25}H_{24}ClN_2O$ $(M+H)^+$ 403.1572, found 403.1560.

(4-Bromophenyl)((1*RS*,3*SR*)-1,3-diphenyltetrahydro-1*H*,5*H*-pyrazolo[1,2-*a*]pyrazol-2-yl)methanone (3d), yield 290 mg (65%), yellow solid, m.p. 133-135 °C. R_f 0.55 (hexane:ethyl acetate 2:1). IR: ν = 2863, 1661 s, 1580 s, 1488, 1453, 1460, 1345, 1290, 1244, 1203, 1180, 1105 s, 1071 s, 1007 s, 747, 699 s cm^{-1}. ^{1}H NMR (400 MHz, DMSO-d_6): δ 7.50-7.46 (m, 2H, Ar), 7.41-7.36 (m, 4H, Ar), 7.35-7.21 (m, 8H, Ar), 4.39 (d, 2H, *J* 7.4 Hz, CH), 4.23 (t, *J* 7.4 Hz, 1H, CH), 3.11-2.96 (m, 4H, CH_2), 2.35-2.23 (m, 1H, CH_2), 1.95-1.84 (m, 1H, CH_2). ^{13}C NMR (100 MHz, DMSO-d_6): δ 199.0 (CO), 142.5 (2C), 135.7 (C), 132.1 (2CH), 130.7 (2CH), 128.9 (4CH), 128.4 (C), 127.8 (2CH), 127.7 (4CH), 71.6 (2CH), 68.8 (CH), 50.8 (2CH_2), 23.9 (CH_2).

HRMS (ESI): *m/z* calcd. for $C_{25}H_{24}BrN_2O$ $(M+H)^+$ 447.1066, found 447.1067.

((1*RS*,3*SR*)-1,3-Diphenyltetrahydro-1*H*,5*H*-pyrazolo[1,2-*a*]pyrazol-2-yl)(4-methoxyphenyl)-methanone (3e), yield 240 mg (60%), light yellow solid, m.p. 115-117 °C. R_f 0.51 (hexane:ethyl acetate 2:1). IR: ν = 2973, 2934, 1658 s, 1599 s, 1573, 1510, 1490, 1420, 1353, 1257 s, 1215, 1180 s, 1123, 1015, 863, 703 s cm^{-1}. ^{1}H NMR (400 MHz, $CDCl_3$): δ 7.55-7.48 (m, 6H, Ar), 7.37-7.25 (m, 6H, Ar), 6.74-6.70 (m, 2H, Ar), 4.50 (d, 2H, *J* 7.3 Hz, CH), 4.30 (t, *J* 7.3 Hz, 1H, CH), 3.80 (s, 3H, Me), 3.34-3.26 (m, 2H, CH_2), 3.25-3.17 (m, 2H, CH_2), 2.40-2.30 (m, 1H, CH_2), 2.11-2.01 (m, 1H, CH_2). ^{13}C NMR (100 MHz, $CDCl_3$): δ 197.9 (CO), 163.9 (C), 142.8 (2C), 131.1 (2CH), 129.8 (C), 128.9 (4CH), 127.7 (6CH), 114.3 (2CH), 72.0 (2CH), 68.5 (CH), 56.0 (CH_3), 50.8 (2CH_2), 23.9 (CH_2).
HRMS (ESI): *m/z* calcd. for $C_{26}H_{27}N_2O_2$ [(M+H)$^+$] 399.2073, found: 399.2072.
((1*RS*,2*RS*,3*SR*)-1,3-Bis(4-chlorophenyl)tetrahydro-1*H*,5*H*-pyrazolo[1,2-*a*]pyrazol-2-yl)(phenyl)methanone (3f), yield 300 mg (69%), light yellow solid, m.p. 131-132 °C. R_f 0.55 (hexane:ethyl acetate 3:1). IR: ν = 2895, 1668 s, 1595, 1488 s, 1405, 1367, 1276, 1086 s, 1013 s, 900, 832, 788, 711 cm^{-1}. ^{1}H NMR (400 MHz, $CDCl_3$): δ 7.50-7.43 (m, 3H, Ar), 7.41-7.35 (m, 4H, Ar), 7.32-7.25 (m, 6H, Ar), 4.42 (d, 2H, *J* 7.3 Hz, CH), 4.21 (t, *J* 7.3 Hz, 1H, CH), 3.32-3.24 (m, 2H, CH_2), 3.19-3.12 (m, 2H, CH_2), 2.40-2.27 (m, 1H, CH_2), 2.12-2.02 (m, 1H, CH_2). ^{13}C NMR (100 MHz, $CDCl_3$): δ 199.5 (CO), 140.6 (2C), 136.4 (2C), 133.7 (CH), 133.2 (2CH), 133.1 (C), 128.9 (2CH), 128.8 (2CH), 128.7 (4CH), 128.5 (2CH), 71.2 (2CH), 68.9 (CH), 50.9 (2CH_2), 23.8 (CH_2).
HRMS (ESI): *m/z* calcd. for $C_{25}H_{23}Cl_2N_2O$ (M+H)$^+$] 437.1182, found: 437.1179.
((1*RS*,3*SR*)-1,3-Bis(4-chlorophenyl)tetrahydro-1*H*,5*H*-pyrazolo[1,2-*a*]pyrazol-2-yl)(*p*-tolyl)-methanone (3g), yield 230 mg (51%), light yellow solid, m.p. 67-69 °C. R_f 0.50 (hexane:ethyl acetate 3:1). IR: ν = 2917, 1666 s, 1603 s, 1488 s, 1409, 1359, 1269, 1184 s, 1086 s, 1013, 816 cm^{-1}. ^{1}H NMR (400 MHz, $CDCl_3$): δ 7.45-7.35 (m, 6H, Ar), 7.32-7.23 (m, 4H, Ar), 7.20-7.07 (m, 2H, Ar), 4.41 (d, 2H, *J* 7.3 Hz, CH), 4.19 (t, *J* 7.3 Hz, 1H, CH), 3.32-3.24 (m, 2H, CH_2), 3.20-3.13 (m, 2H, CH_2), 2.38-2.28 (m, 1H, CH_2), 2.35 (s, 3H, Me), 2.11-2.03 (m, 1H, CH_2). ^{13}C NMR (100 MHz, $CDCl_3$): δ 199.0 (CO), 144.8 (C), 140.6 (2C), 133.9 (C), 132.8 (2C), 129.2 (2CH), 128.9 (2CH), 128.8 (4CH), 128.7 (4CH), 71.3 (2CH), 68.6 (CH), 50.8 (2CH_2), 23.8 (CH_2), 21.6 (CH_3).
HRMS (ESI): *m/z* calcd. for $C_{26}H_{25}Cl_2N_2O$ (M+H)$^+$ 451.1338, found: 451.1334.
((1*RS*,3*SR*)-1,3-Bis(4-chlorophenyl)tetrahydro-1*H*,5*H*-pyrazolo[1,2-*a*]pyrazol-2-yl)(4-chlorophenyl)methanone (3h), yield 310 mg (66%), yellow solid, m.p. 88-89 °C. R_f 0.45 (hexane:ethyl acetate 3:1). IR: ν = 2882, 1672 s, 1586 s, 1489 s, 1402, 1344, 1283, 1204, 1180, 1089 s, 1011 s, 917, 817 cm^{-1}. ^{1}H NMR (400 MHz, DMSO-d_6): δ 7.50-7.46 (m, 2H, Ar), 7.41-7.33 (m, 10H, Ar), 4.40 (d, 2H, *J* 7.3 Hz, CH), 4.20 (t, *J* 7.3 Hz, 1H, CH), 3.13-3.05 (m, 2H,

CH_2), 3.05-2.95 (m, 2H, CH_2), 2.35-2.25 (m, 1H, CH_2), 1.95-1.85 (m, 1H, CH_2). ^{13}C NMR (100 MHz, DMSO-d$_6$): δ 198.5 (CO), 141.4 (2C), 139.3 (C), 135.3 (C), 132.3 (2C), 130.7 (2CH), 129.7 (2CH), 129.2 (4CH), 128.9 (4CH), 70.6 (2CH), 68.3 (CH), 50.7 (2CH$_2$), 23.8 (CH$_2$).
HRMS (ESI): *m/z* calcd. for $C_{25}H_{22}Cl_3N_2O$ (M+H)$^+$ 471.0798, found: 471.0781.

((1*RS*,3*SR*)-1,3-Bis(4-chlorophenyl)tetrahydro-1*H*,5*H*-pyrazolo[1,2-*a*]pyrazol-2-yl)(4-methoxyphenyl)methanone (3i), yield 230 mg (50%), yellow wax, R_f 0.50 (hexane:ethyl acetate 3:1). IR: ν = 2962, 1664 s, 1599 s, 1573, 1510, 1489 s, 1420, 1259 s, 1168, 1088 s, 1013, 813 cm^{-1}. ^{1}H NMR (400 MHz, $CDCl_3$): δ 7.50-7.42 (m, 2H, Ar), 7.37-7.31 (m, 4H, Ar), 7.30-7.22 (m, 4H, Ar), 6.76-6.70 (m, 2H, Ar), 4.41 (d, 2H, *J* 7.4 Hz, CH), 4.16 (t, *J* 7.4 Hz, 1H, CH), 3.82 (s, 3H, CH_3), 3.33-3.23 (m, 2H, CH_2), 3.20-3.14 (m, 2H, CH_2), 2.40-2.30 (m, 1H, CH_2), 2.10-2.00 (m, 1H, CH_2). ^{13}C NMR (100 MHz, $CDCl_3$): δ 197.6 (CO), 164.0 (C), 140.8 (2C), 133.2 (2C), 131.2 (2CH), 129.5 (C), 128.8 (4CH), 128.7 (4CH), 113.7 (2CH), 71.3 (2CH), 68.5 (CH), 55.5 (CH_3), 50.9 (2CH$_2$), 23.8 (CH$_2$).
HRMS (ESI): *m/z* calcd. for $C_{26}H_{25}Cl_2N_2O_2$ (M+H)$^+$ 467.1288, found: 467.1291.

((1*RS*,3*SR*)-1,3-Bis(p-tolyl)tetrahydro-1*H*,5*H*-pyrazolo[1,2-*a*]pyrazol-2-yl)(phenyl)-methanone (3j), yield 225 mg (57%), light yellow solid, m.p. 84-86 °C. R_f 0.55 (hexane:ethyl acetate 3:1). IR: ν = 2923, 1668 s, 1595, 1511, 1447, 1340, 1178, 1100, 1019, 814, 792 cm^{-1}. ^{1}H NMR (400 MHz, $CDCl_3$): δ 7.55-7.49 (m, 1H, Ar), 7.46-7.41 (m, 2H, Ar), 7.33-7.25 (m, 6H, Ar), 7.14-7.07 (m, 4H, Ar), 4.32 (d, 2H, *J* 7.3 Hz, CH), 4.24 (t, *J* 7.3 Hz, 1H, CH), 3.09-2.98 (m, 4H, CH_2), 2.33-2.22 (m, 1H, CH_2), 2.27 (s, 6H, Me), 1.94-1.84 (m, 1H, CH_2). ^{13}C NMR (100 MHz, DMSO-d$_6$): δ 200.1 (CO), 139.7 (2C), 136.9 (C), 136.8 (2C), 134.0 (CH), 129.4 (4CH), 129.1 (2CH), 128.6 (2CH), 127.6 (4CH), 71.7 (2CH), 68.8 (CH), 50.8 (2CH$_2$), 23.9 (CH$_2$), 21.2 (2CH$_3$).
HRMS (ESI): *m/z* calcd. for $C_{26}H_{29}N_2O$ (M+H)$^+$ 397.2275, found: 397.2282.

((1*RS*,3*SR*)-1,3-Bis(p-tolyl)tetrahydro-1*H*,5*H*-pyrazolo[1,2-*a*]pyrazol-2-yl)(4-chlorophenyl)methanone (3k), yield 50 mg (12%), light yellow solid, m.p. 98-99 °C. R_f 0.50 (hexane:ethyl acetate 3:1). IR: ν = 2950, 1673 s, 1586 s, 1512, 1400, 1276, 1209, 1176, 1090 s, 998, 802 cm^{-1}. ^{1}H NMR (400 MHz, DMSO-d$_6$): δ 7.43 (d, 2H, *J* 8.7 Hz, Ar), 7.35 (d, 2H, *J* 8.7 Hz, Ar), 7.26 (d, 4H, *J* 8.0 Hz, Ar), 7.10 (d, 4H, *J* 8.0 Hz, Ar), 4.32 (d, 2H, *J* 7.4 Hz, CH), 4.21 (t, *J* 7.4 Hz, 1H, CH), 3.09-2.95 (m, 4H, CH_2), 2.30-2.21 (m, 1H, CH_2), 2.28 (s, 6H, Me), 1.93-1.82 (m, 1H, CH_2). ^{13}C NMR (100 MHz, DMSO-d$_6$): δ 199.0 (CO), 139.5 (C), 139.1 (C), 136.9 (2C), 135.5 (2C), 130.5 (2CH), 129.5 (4CH), 129.2 (2CH), 127.6 (4CH), 71.6 (2CH), 68.7 (CH), 50.8 (2CH$_2$), 23.9 (CH$_2$), 21.2 (2CH$_3$).
HRMS (ESI): *m/z* calcd. for $C_{27}H_{28}ClN_2O$ (M+H)$^+$ 431.1885, found: 431.1904.

((1*RS*,3*SR*)-1,3-Bis(4-methoxyphenyl)tetrahydro-1*H*,5*H*-pyrazolo[1,2-*a*]pyrazol-2-yl)(phenyl)methanone (**3l)**, yield 180 mg (42%), yellow solid, m.p. 134-135 °C. R_f 0.45 (hexane:ethyl acetate 3:1). IR: ν = 2934, 1670 s, 1609, 1511 s, 1444, 1296, 1248 s, 1171, 1037, 828 cm^{-1}. ^{1}H NMR (400 MHz, DMSO-d_6): δ 7.53-7.48 (m, 1H, Ar), 7.45-7.40 (m, 2H, Ar), 7.37-7.28 (m, 6H, Ar), 6.90-6.82 (m, 4H, Ar), 4.30 (d, 2H, *J* 7.3 Hz, CH), 4.22 (t, *J* 7.3 Hz, 1H, CH), 3.71 (s, 6H, OMe), 3.08-2.97 (m, 4H, CH_2), 2.33-2.22 (m, 1H, CH_2), 1.94-1.84 (m, 1H, CH_2). ^{13}C NMR (100 MHz, DMSO-d_6): δ 200.1 (CO), 158.9 (2C), 136.9 (C), 134.6 (2C), 134.0 (CH), 129.1 (2CH), 128.9 (4CH), 128.7 (2CH), 114.3 (4CH), 71.4 (2CH), 68.9 (CH), 55.5 (2CH_3), 50.7 (2CH_2), 23.9 (CH_2).
HRMS (ESI): *m/z* calcd. for $C_{27}H_{29}N_2O_3$ $(M+H)^+$ 429.2173, found: 429.2166.

((1*RS*,2*RS*,3*SR*)-1-(4-Chlorophenyl)-3-phenyltetrahydro-1*H*,5*H*-pyrazolo[1,2-*a*]pyrazol-2-yl)(phenyl)methanone (**3m)**.

a) The mixture of DABCH **1a** (210 mg, 1.3 mmol), chalcone **2f** (242 mg, 1 mmol), and 2 drops of $BF_3{\cdot}OEt_2$ in 1.5 g of IL [(bmim)(Cl)] was heated at 90 °C during 9 hours with stirring. After cooling, CH_2Cl_2 (5 mL) followed by 10 mL of diethyl ether were added and the resulting mixture was stirred. The organic layer was decanted from IL and the procedure was repeated two times. Then, the solvent was evaporated and the products were isolated by column chromatography on silica gel to obtain adduct **3m**; yield 150 mg (37%), light yellow solid. R_f 0.42 (hexane:EtOAc 2:1). IR: ν = 2894, 1670 s, 1597, 1489, 1449, 1369, 1252, 1206, 1087 s, 1014 s, 820, 786 cm^{-1}. ^{1}H NMR (400 MHz, DMSO-d_6): δ 7.54-7.49 (m, 1H, Ar), 7.45-7.39 (m, 4H, Ar), 7.38-7.33 (m, 4H, Ar), 7.31-7.21 (m, 5H, Ar), 4.45 (d, 1H, *J* 7.0 Hz, CH), 4.35 (d, 1H, *J* 7.6 Hz, CH), 4.17 (dd, *J* 7.6, 7.0 Hz, 1H, CH), 3.13-2.96 (m, 4H, CH_2), 2.36-2.26 (m, 1H, CH_2), 1.97-1.86 (m, 1H, CH_2). ^{13}C NMR (100 MHz, DMSO-d_6): δ 199.6 (CO), 142.2 (C), 142.1 (C), 136.7 (C), 134.1 (2CH), 132.1 (C), 129.6 (2CH), 129.2 (2CH), 128.9 (2CH), 128.8 (2CH), 128.7 (2CH), 127.9 (2CH), 71.9 (CH), 70.6 (CH), 68.7 (CH), 50.8 (CH_2), 50.7 (CH_2), 23.9 (CH_2).
HRMS (ESI): *m/z* calcd. for $C_{25}H_{23}ClN_2O$ $[(M+H)^+]$ 403.1572, found: 403.1574.

b) using the same procedure starting with DABCH **1b** and chalcone **2a** we received adduct **3m** with 10% yield.

The characteristics corresponded to that described in the literature [4].

2-(4-Bromophenyl)-9-(4-chlorophenyl)tetrahydro-5*H*-pyrazolo[1,2-*a*]pyrrolo[3,4-*c*]pyrazole-1,3(2*H*,3a*H*)-dione (6).

a) The mixture of diazabicyclohexane **1c** (213 mg, 1.1 mmol) and 4-bromophenylmaleimide (**5**, 253 mg, 1 mmol) in 4 mL toluene was heated at 110 °C during 3 hours. Then, toluene was removed under reduced pressure and the product was obtained by column chromatography on silica gel, using gradient elution with hexane/ethyl acetate with a yield of 115 mg (26%).

b) A mixture of the adduct **3g** (100 mg, 0.23mmol) and imide **5** (90 mg (0.36 mmol) in 2 mL of toluene was heated at 110 °C under microwave irradiation during 3 hours. Then, toluene was removed under reduced pressure and the products were obtained by column chromatography on silica gel, using gradient elution with hexane/ethyl acetate with a yield of 30 mg (30%). Light yellow solid, m.p. 147-148 °C. R_f 0.63 (hexane: ethyl acetate 2:1). IR: ν = 1716 s, 1490, 1392, 1202, 1090, 1013, 786 cm^{-1}. ^1H NMR (400 MHz, $CDCl_3$): δ 7.65-7.61 (m, 2H, Ar), 7.58-7.53 (m, 2H, Ar), 7.44-7.38 (m, 2H, Ar), 7.29-7.24 (m, 2H, Ar), 4.69-4.57 (m, 1H), 4.04-3.99 (m, 1H), 3.98-3.91 (m, 1H), 3.51-3.43 (m, 1H), 3.31-3.19 (m, 1H), 2.73-2.60 (m, 2H), 2.35-2.20 (m, 2H). ^{13}C NMR (100 MHz, DMSO-d_6): δ 175.8 (CO), 174.8 (CO), 132.9 (C), 139.1 (C), 132.5 (2CH), 131.8 (C), 130.5 (2CH), 129.6 (2CH), 128.9 (2CH), 122.0 (C), 67.4 (2CH), 66.8 (CH), 51.0 (CH_2), 46.7 (CH_2), 25.3 (CH_2).

HRMS (ESI): *m/z* calcd. for $C_{20}H_{18}BrClN_3O_2$ $(M+H)^+$ 446.0266 , found: 446.0256.

The compound **6** was described earlier as the mixture of diastereomers [5].

2,3-Bis(4-chlorophenyl)tetrahydropyrazolo[1,2-*a*][1,2,4]triazole-1(5*H*)-one (**8**). A mixture of the adduct **3g** (90 mg (0.20 mmol) and 4-chlorophenylisocyanate (**7**, 65 mg (0.42 mmol) in 2 mL of toluene was heated at 110 °C under microwave irradiation during 3 hours. Then, toluene was removed under reduced pressure and the product was obtained by column chromatography on silica gel, using gradient elution with hexane/ethyl acetate with a yield of 60 mg (82%). Light yellow solid, m.p. 158-159 °C. R_f 0.54 (hexane: ethyl acetate 2:1). IR: ν = 2839, 1692 s, 1600, 1497 s, 1423, 1387 s, 1293, 1091, 826 cm^{-1}. ^1H NMR (400 MHz, DMSO-d_6): δ 7.64-7.52 (m, 2H, Ar), 7.46-7.33 (m, 6H, Ar), 6.45 (s, 1H), 3.80-3.67 (m, 1H), 3.13-2.99 (m, 1H), 2.64-2.50 (m, 2H), 2.17-2.06 (m, 1H), 2.05-1.95 (m, 1H). ^{13}C NMR (100 MHz, DMSO-d_6): δ 161.2 (CO), 137.4 (2C), 133.6 (C), 129.3 (2CH), 129.1 (4CH), 127.8 (C), 120.8 (2CH), 76.1 (CH), 52.4 (CH_2), 44.3 (CH_2), 25.2 (CH_2).

HRMS (ESI): *m/z* calcd. for $C_{17}H_{16}Cl_2N_3O$ $(M+H)^+$ 348.0665, found: 348.0664.

2-(4-Bromophenyl)-3-(4-chlorophenyl)tetrahydro-1*H*,5*H*-pyrazolo[1,2-*a*][1,2,4]triazole-1-thione (**10**). A mixture of the adduct **3g** (100 mg (0.22 mmol) and 4-bromophenylisothiocyanate (**9**, 107 mg (0.50 mmol) in 2 mL of toluene was heated at 110 °C under microwave irradiation during 3 hours. Then, toluene was removed under reduced pressure and the product was obtained by column chromatography on silica gel, using gradient elution with hexane/ethyl acetate with a yield of 50 mg (56%). Light yellow solid, m.p. 152-153 °C. R_f 0.50 (hexane: ethyl acetate 2:1). IR: ν = 1492 s, 1420, 1386 s, 1317, 1257, 1090, 1042, 1007, 831 cm^{-1}. ^1H NMR (400 MHz, DMSO-d_6): δ 7.53-7.44 (m, 2H, Ar), 7.36-7.20 (m, 6H, Ar), 5.92 (s, 1H), 4.56-4.42 (m, 1H), 3.66-3.55 (m, 1H), 3.36-3.26 (m, 2H), 2.81-2.71 (m, 1H), 2.34-2.21 (m, 1H). ^{13}C NMR (100

MHz, DMSO-d_6): δ 184.2 (CS), 137.0 (C), 135.4 (C), 134.8 (C), 132.4 (2CH), 129.2 (2CH), 128.5 (2CH), 127.9 (2CH), 121.0 (C), 83.7 (CH), 52.0 (CH_2), 46.9 (CH_2), 24.7 (CH_2).

HRMS (ESI): *m/z* calcd. for $C_{17}H_{16}BrClN_3S$ $(M+H)^+$ 407.9931, found: 407.9747.

X-ray analysis of compound 3e and 3i

Crystallographic data:

A single crystal suitable for shooting was selected and studied by X-ray diffraction. Crystal **3e** ($C_{26}H_{26}N_2O_2$, M = 398.49 g/mol) monoclinic, space group $P2_1/n$, a = 10.0306(5) Å, b = 9.7294(5) Å, c = 21.8681(10) Å, α = 90, β = 98.026(5)°, γ = 90, V = 2113.24(18) Å3, Z = 4 (Z'= 1), T = 99.99(10) K, μ(MoKα) = 0.079 mm^{-1}, D_{calc} = 1.252 g/cm^3, 14414 reflections measured (5.63° ≤ 2Θ ≤ 62.192°), 5953 unique (Rint = 0.0289, Rsigma = 0.0424) which were used in all calculations, ρ_{calc} g/cm^3 =1,252, F (000) = 848.0. The final R1 was 0.0512 (I > 2σ(I)) and wR2 was 0.1281.

X-ray diffraction studies of the crystal of **3e** were performed on an Xcalibur Eos diffractometer using monochromated MoKα (λ = 0.71073) irradiation, and 5953 independent reflections [Rint = 0.0289] were used in further refinement. The structure was solved by direct methods and refined by the full-matrix least squares technique against F2 in the anisotropic-isotropic approximation. Final R-factor – 5.11%.

The primary processing of the experimental data was performed using the CrysAlisPro software package (Agilent Technologies).

The structure of the adduct was solved by direct methods using the SHELX program (Sheldrick, G.M. (2015). Acta Cryst. A71, C71, 3-8) incorporated the Olex2 software package.

Single crystal of **3i** ($C_{27}H_{28}Cl_2N_2O_2$, M = 483.41g/mol) monoclinic, space group $P2_1/c$, a = 9.38230(10) Å, b = 18.1841(2) Å, c = 13.9214(2) Å, α = 90, β = 91.2770(10), γ = 90, V = 2374.52(5) Å3, Z = 4 (Z' = 1), T = 117.6(3) K, μ(CuKα) = 2.675 mm^{-1}, D_{calc} = 1.352 g/cm^3, 23751 reflections measured (8° ≤ 2Θ ≤ 145.944°), 4719 unique (Rint = 0.0418, Rsigma = 0.0234) which were used in all calculations, ρ_{calc} g/cm^3 = 1,352, F (000) = 1016.0. The final R1 was 0.0372 (I > 2σ(I)) and wR2 was 0.0968.

X-ray diffraction studies of methanol solvate **3i**·MeOH were performed on a SuperNova, Dual, Atlas diffractometer using monochromated CuKα (λ = 1.54184 Å) irradiation, and 4719 independent reflections [Rint = 0.0418] were used in further refinement. The structure was solved by direct methods and refined by the full-matrix leastsquares technique against F2 in the anisotropic-isotropic approximation. Final R-factor – 3.72%.

The primary processing of the experimental data was performed using the CrysAlisPro software package (Agilent Technologies).

The structure of the adduct was solved by direct methods using the SHELX program (Sheldrick, G.M. (2015). Acta Cryst. A71, C71, 3-8) incorporated the Olex2 software package.

Accession Codes CCDC **1977989** (**3e**) and **1977990** (**3i**) contain the supplementary crystallographic data for this paper. These data can be obtained free of charge via www.ccdc.cam.ac.uk/data_request/cif, or by emailing data_request@ccdc.cam.ac.uk, or by contacting The Cambridge Crystallographic Data Centre, 12 Union Road, Cambridge CB2 1EZ, UK; fax: +44 1223 336033.

References

1. Koptelov, Yu. B.; Kim, M. Kh.; Molchanov, A. P.; Kostikov, R. R. *Russ. J. Org. Chem.*, **1999**, *35*, 110–118.
2. Molchanov, A. P.; Sipkin, D. I.; Koptelov, Yu. B.; Kostikov, R. R.; *Synlett* **2000,** 1779–1780. doi: 10.1055/s-2000-8690
3. Molchanov, A. P.; Sipkin, D. I.; Koptelov, Yu. B.; Kostikov, R. R. *Eur. J. Org. Chem.* **2002,** 453–456. doi:10.1002/1099-0690(20022)2002:3<453::AID-EJOC453>3.0.CO;2-R
4. Hu, H.; Xu, J.; Wang, F.; Dong, S.; Liu, X.; Feng, X. *Org. Lett.* **2020,** *22,* 93−97. doi:10.1021/acs.orglett.9b04007
5. Molchanov, A. P.; Sipkin, D. I.; Koptelov, Yu. B.; Kostikov, R. R. *Russ. J. Org. Chem.*, **2001**, *37*, 841–851. doi:10.1023/A:1012465732305

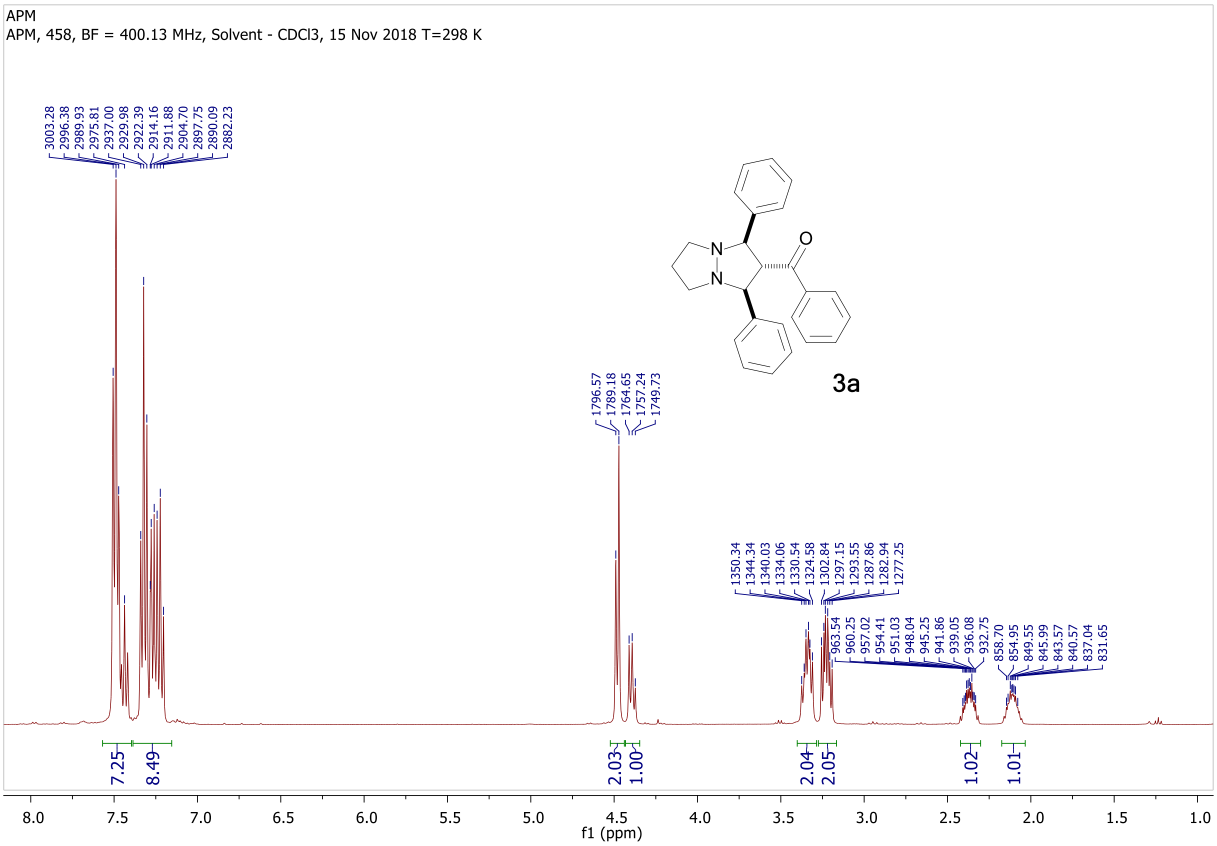
APM
APM, 458, BF = 400.13 MHz, Solvent - CDCl3, 15 Nov 2018 T=298 K
3a
f1 (ppm)

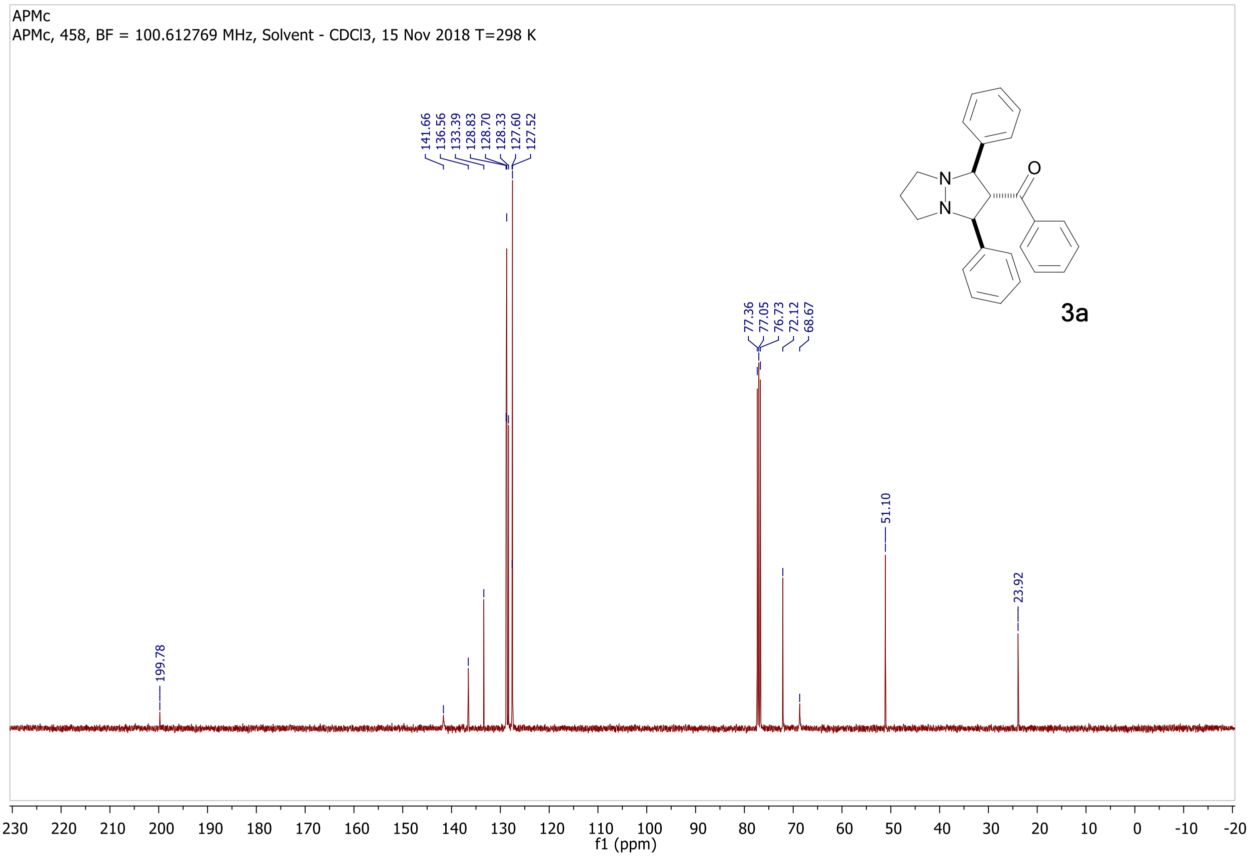
APMc
APMc, 458, BF = 100.612769 MHz, Solvent - CDCl3, 15 Nov 2018 T=298 K
3a
f1 (ppm)

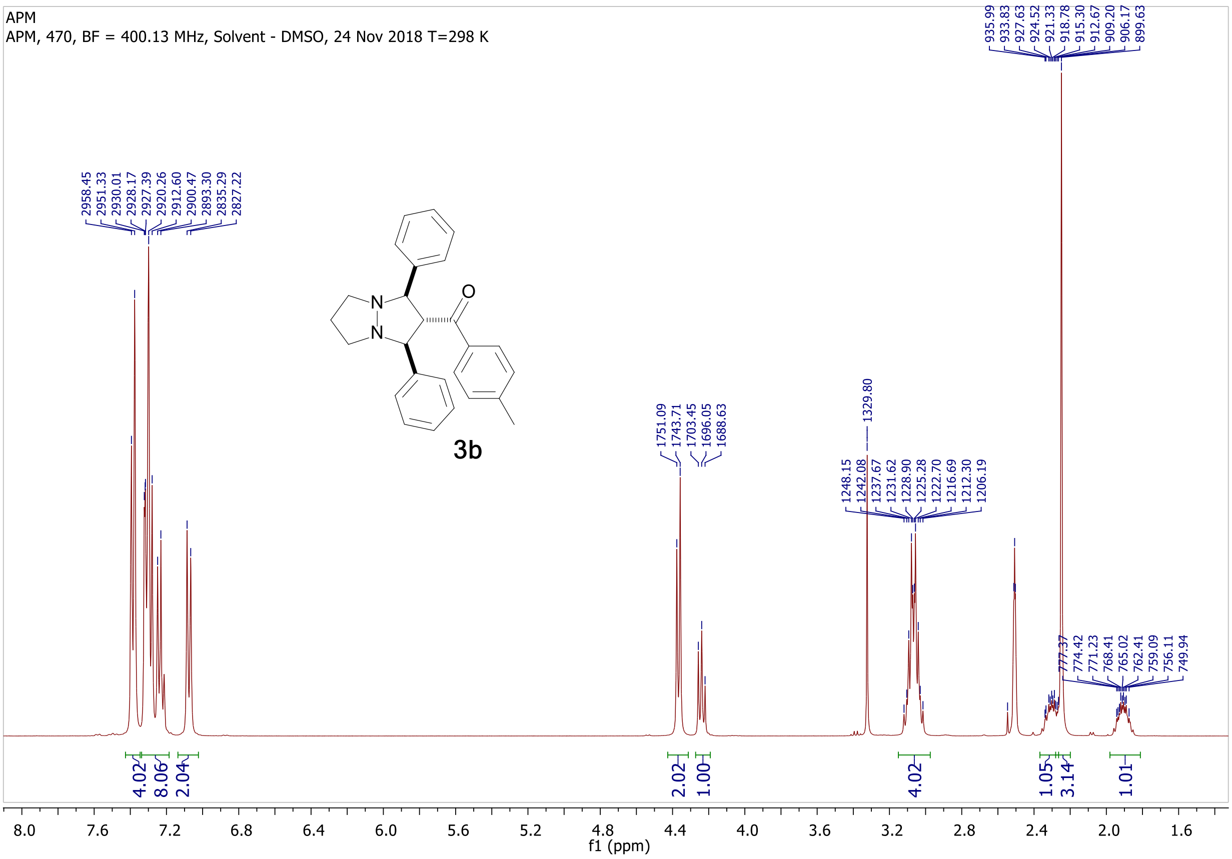

APM
APM, 470, BF = 400.13 MHz, Solvent - DMSO, 24 Nov 2018 T=298 K
3b
f1 (ppm)

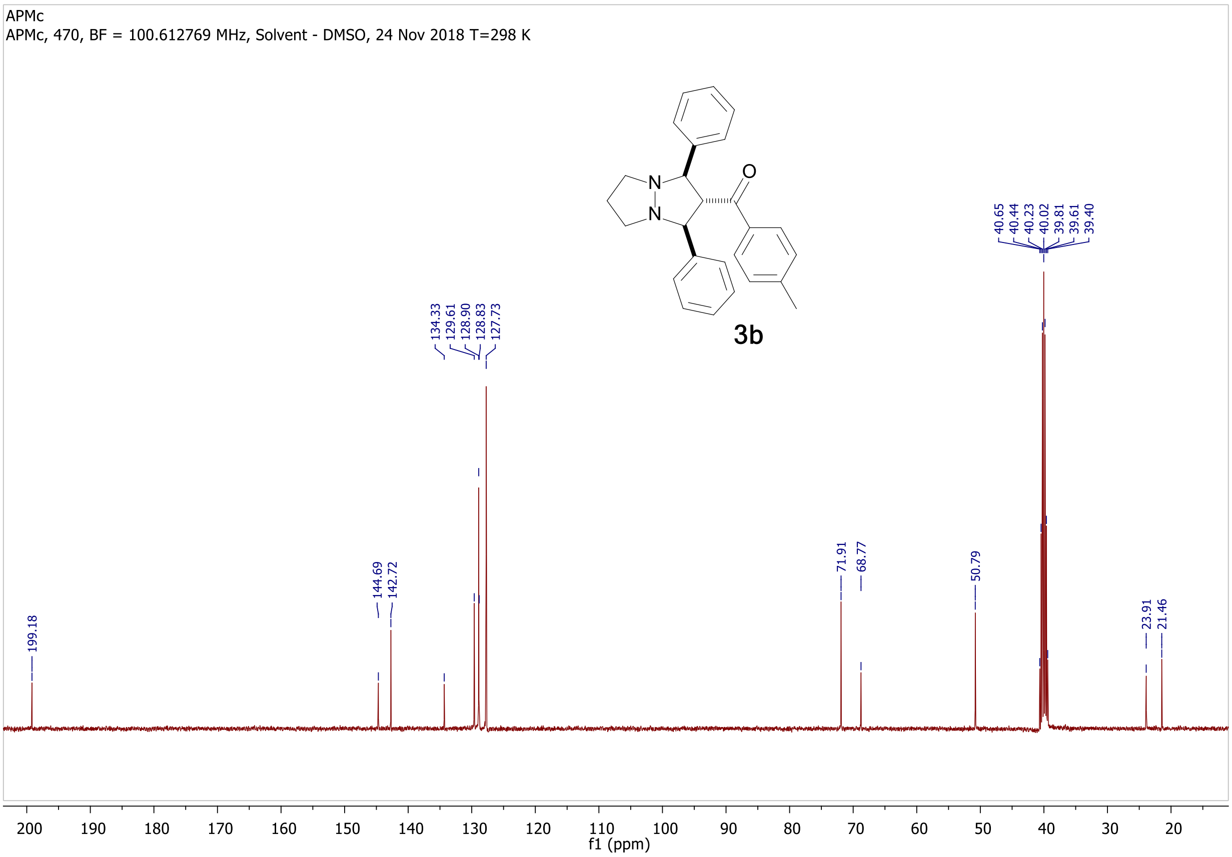

APMc
APMc, 470, BF = 100.612769 MHz, Solvent - DMSO, 24 Nov 2018 T=298 K
3b
f1 (ppm)

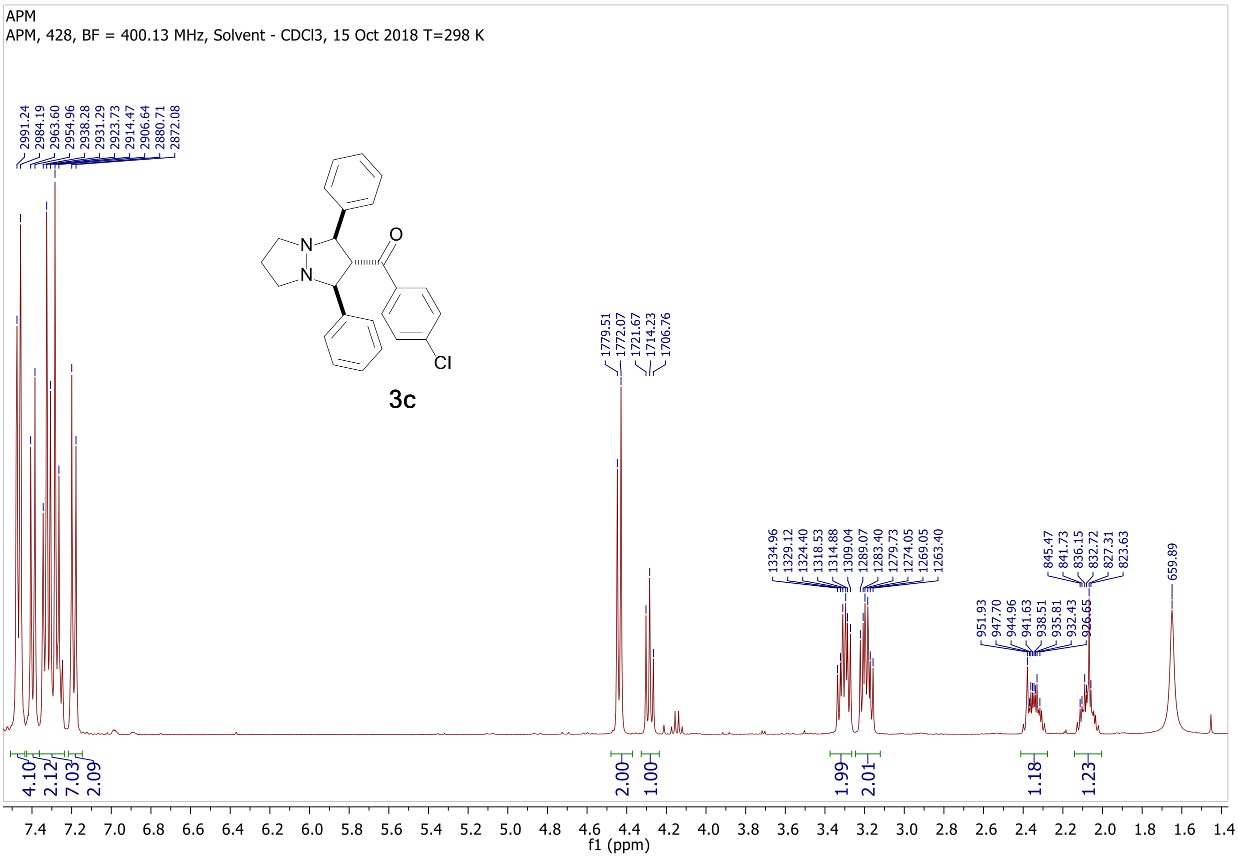
APM
APM, 428, BF = 400.13 MHz, Solvent - CDCl3, 15 Oct 2018 T=298 K
2991.24
2984.19
2963.60
2954.96
2938.28
2931.29
2923.73
2914.47
2906.64
2880.71
2872.08
1779.51
1772.07
1721.67
1714.23
1706.76
1334.96
1329.12
1324.40
1318.53
1314.88
1309.04
1289.07
1283.40
1279.73
1274.05
1269.05
1263.40
951.93
947.70
944.96
941.63
938.51
935.81
932.43
926.65
845.47
841.73
836.15
832.72
827.31
823.63
659.89
O
N
N
Cl
3c
4.10
2.12
7.03
2.09
2.00
1.00
1.99
2.01
1.18
1.23
f1 (ppm)

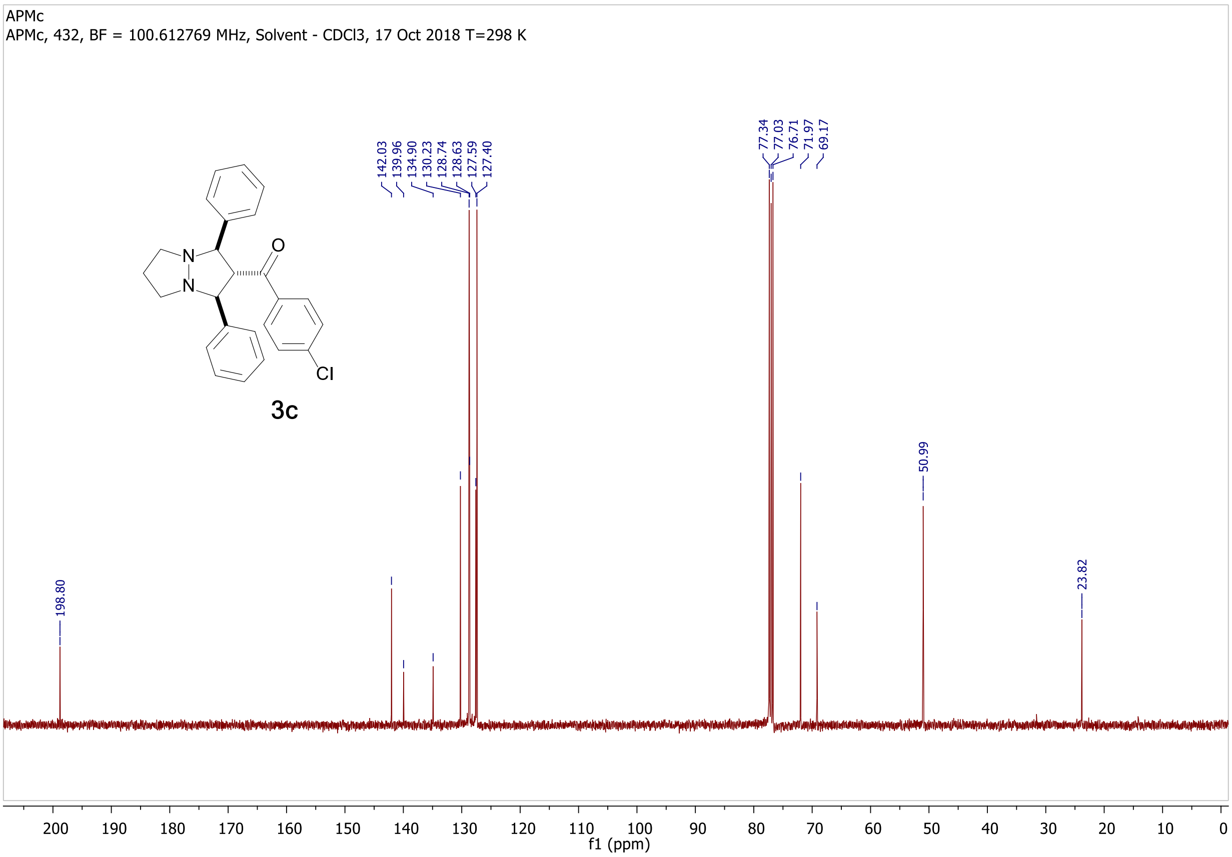
APMc
APMc, 432, BF = 100.612769 MHz, Solvent - CDCl3, 17 Oct 2018 T=298 K
142.03
139.96
134.90
130.23
128.74
128.63
127.59
127.40
77.34
77.03
76.71
71.97
69.17
198.80
50.99
23.82
O
N
N
Cl
3c
f1 (ppm)

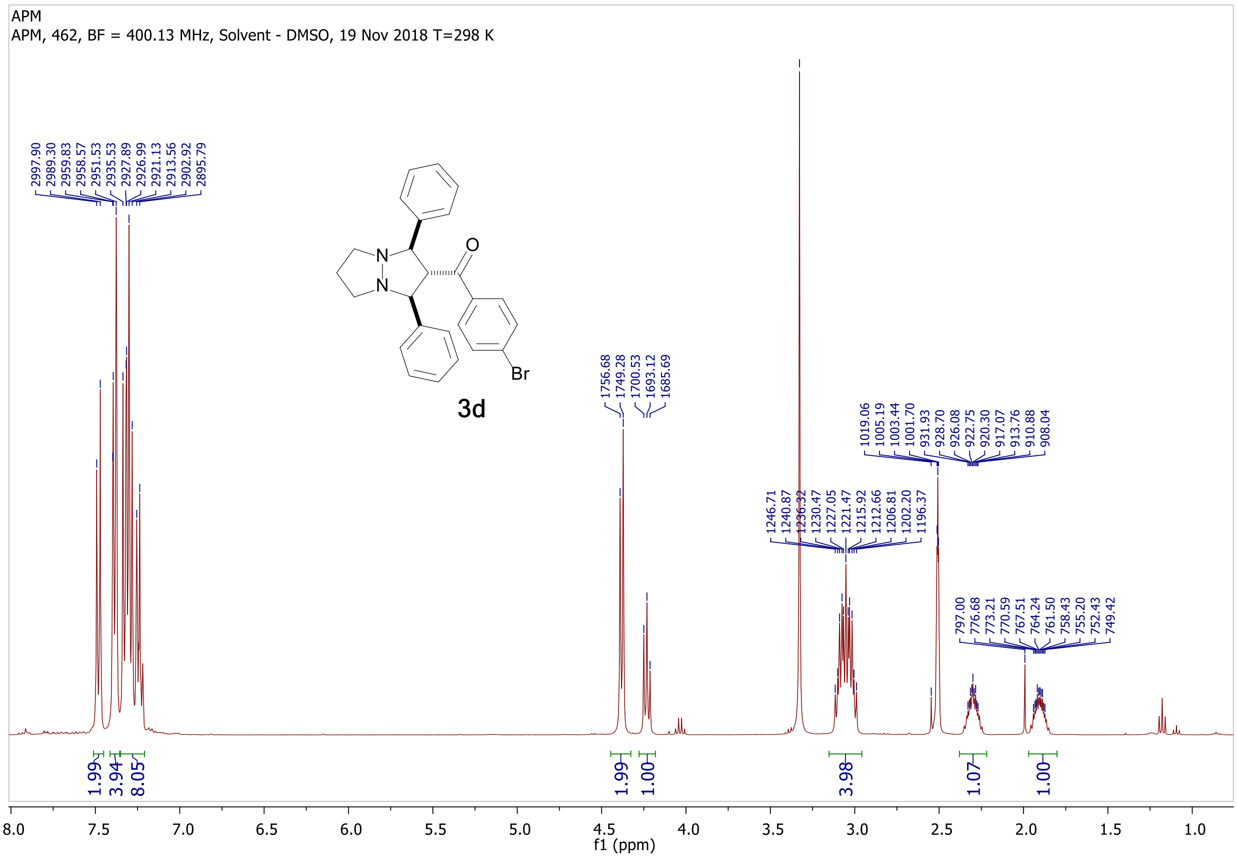
APM
APM, 462, BF = 400.13 MHz, Solvent - DMSO, 19 Nov 2018 T=298 K
2997.90
2989.30
2959.83
2958.57
2951.53
2935.53
2927.89
2926.99
2921.13
2913.56
2902.92
2895.79
1756.68
1749.28
1700.53
1693.12
1685.69
1246.71
1240.87
1236.32
1230.47
1227.05
1221.47
1215.92
1212.66
1206.81
1202.20
1196.37
1019.06
1005.19
1003.44
1001.70
931.93
928.70
926.08
922.75
920.30
917.07
913.76
910.88
908.04
797.00
776.68
773.21
770.59
767.51
764.24
761.50
758.43
755.20
752.43
749.42
N
N
O
Br
3d
1.99
3.94
8.05
1.99
1.00
3.98
1.07
1.00
8.0
7.5
7.0
6.5
6.0
5.5
5.0
4.5
4.0
3.5
3.0
2.5
2.0
1.5
1.0
f1 (ppm)

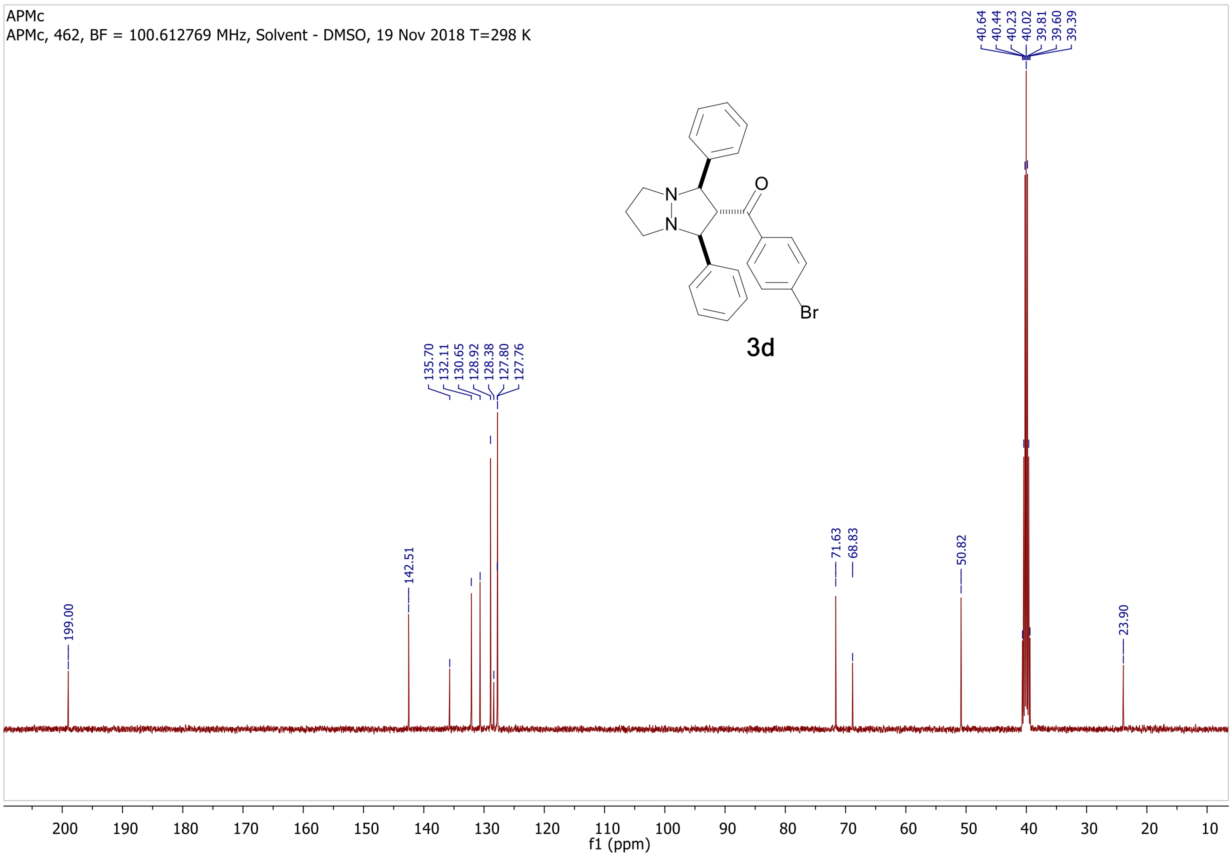
APMc
APMc, 462, BF = 100.612769 MHz, Solvent - DMSO, 19 Nov 2018 T=298 K
40.64
40.44
40.23
40.02
39.81
39.60
39.39
N
N
O
Br
3d
135.70
132.11
130.65
128.92
128.38
127.80
127.76
199.00
142.51
71.63
68.83
50.82
23.90
200
190
180
170
160
150
140
130
120
110
100
90
80
70
60
50
40
30
20
10
f1 (ppm)

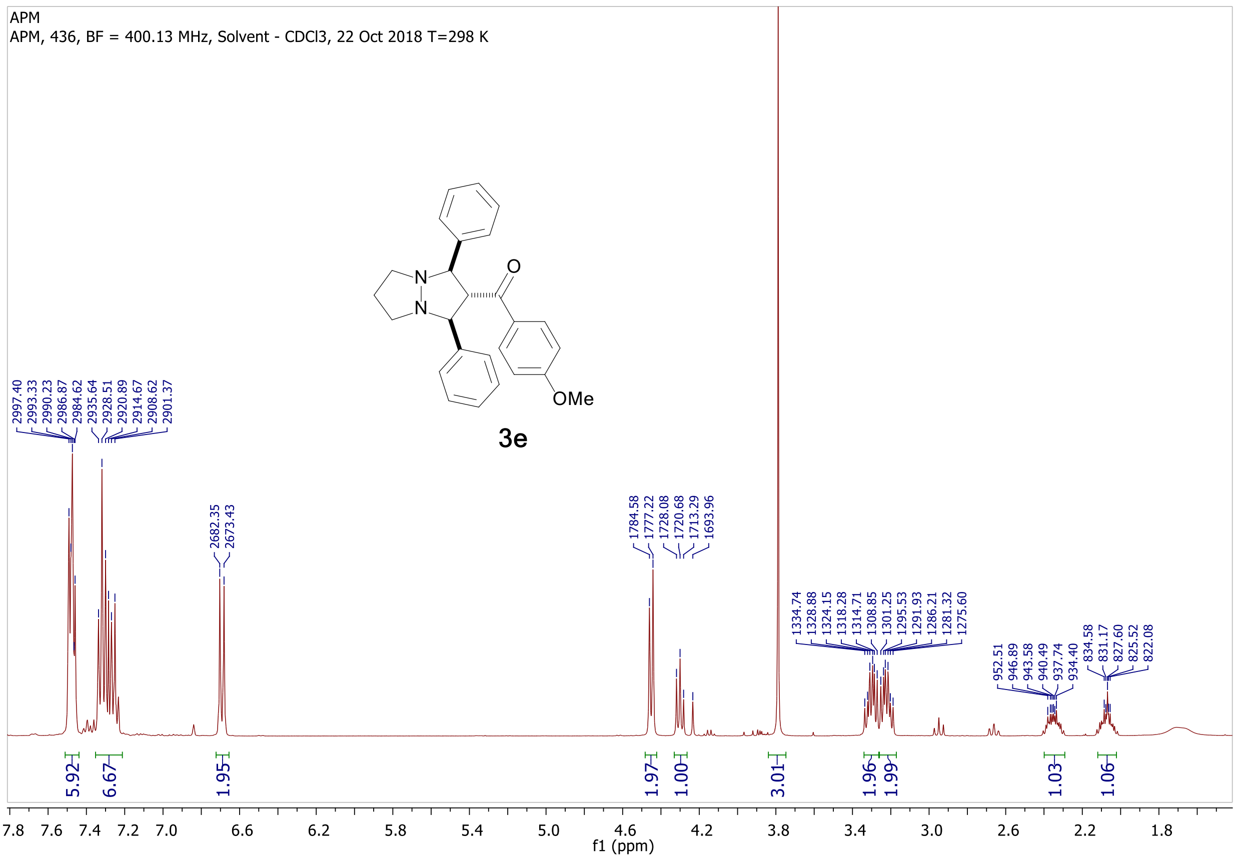
APM
APM, 436, BF = 400.13 MHz, Solvent - CDCl3, 22 Oct 2018 T=298 K
3e
OMe
f1 (ppm)

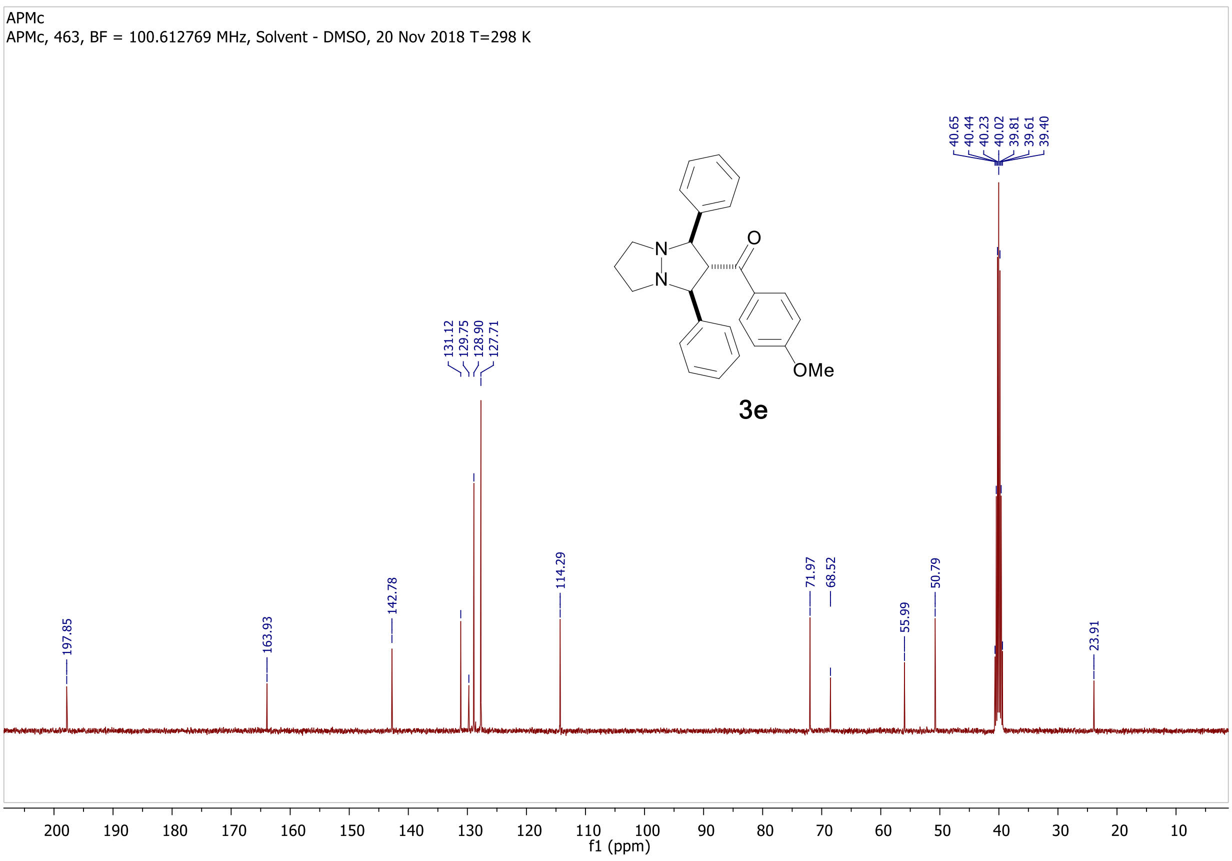
APMc
APMc, 463, BF = 100.612769 MHz, Solvent - DMSO, 20 Nov 2018 T=298 K
3e
OMe
f1 (ppm)

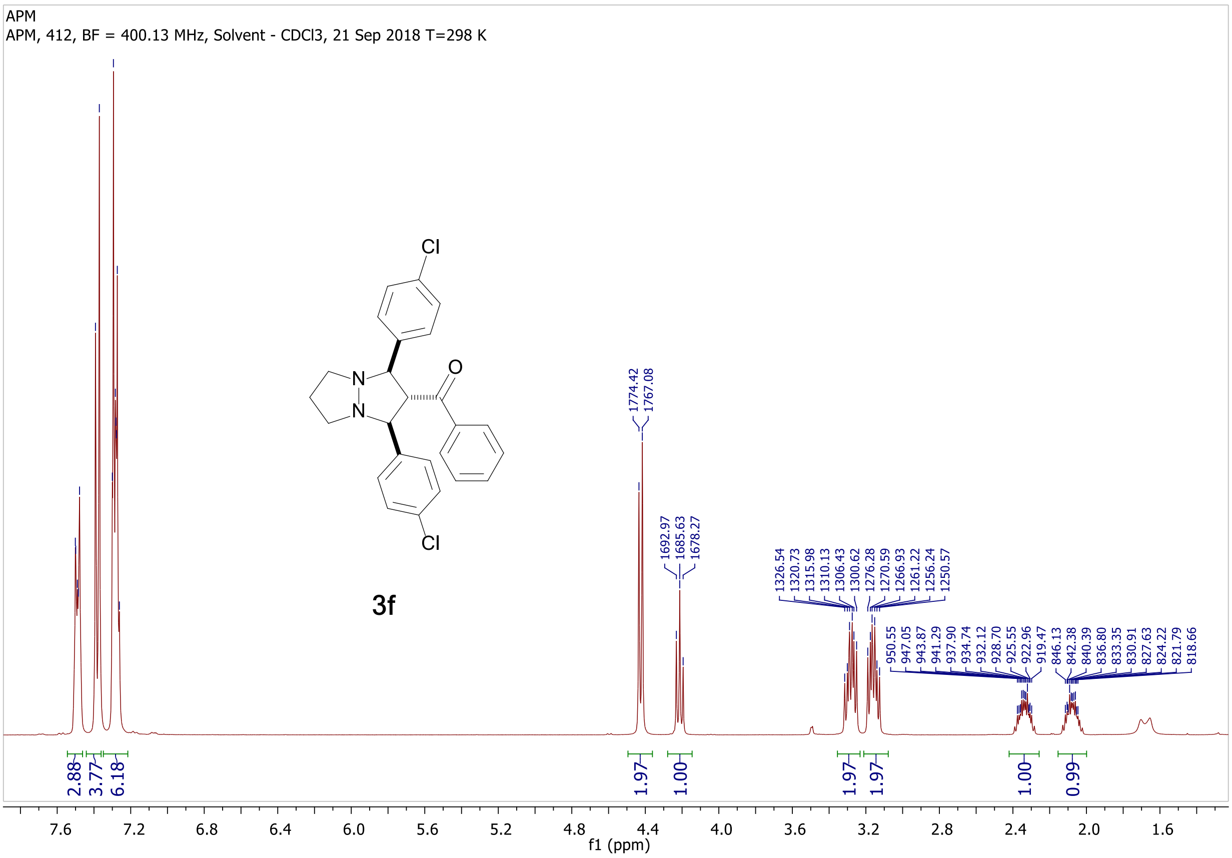

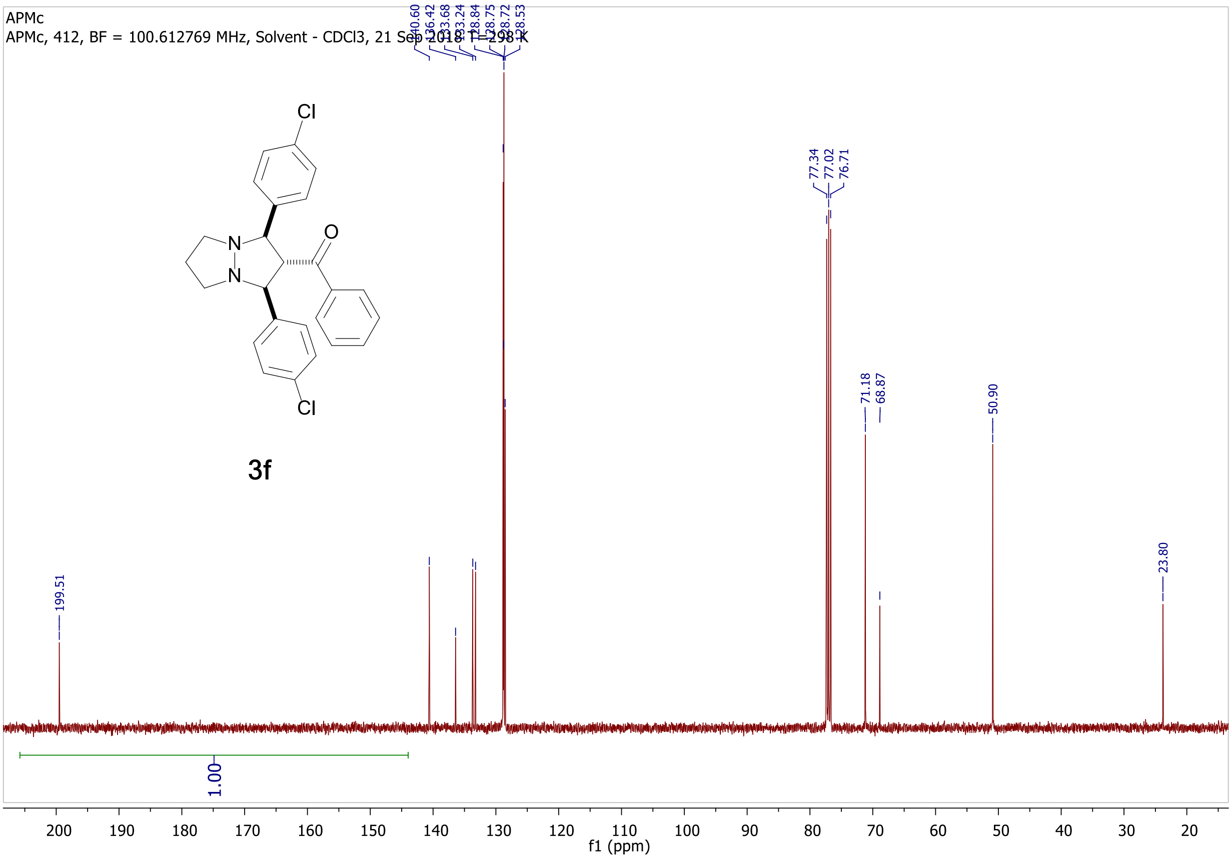

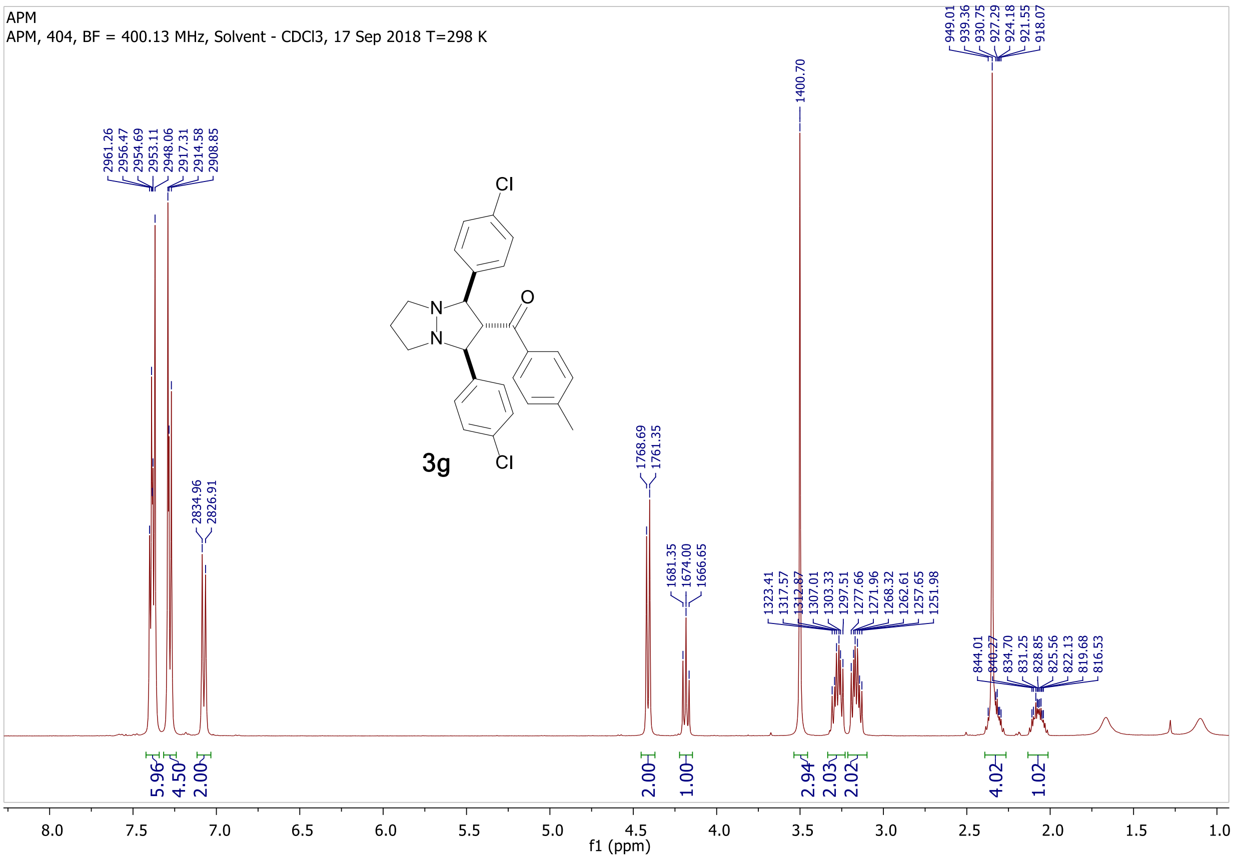
APM
APM, 404, BF = 400.13 MHz, Solvent - CDCl3, 17 Sep 2018 T=298 K
3g
f1 (ppm)

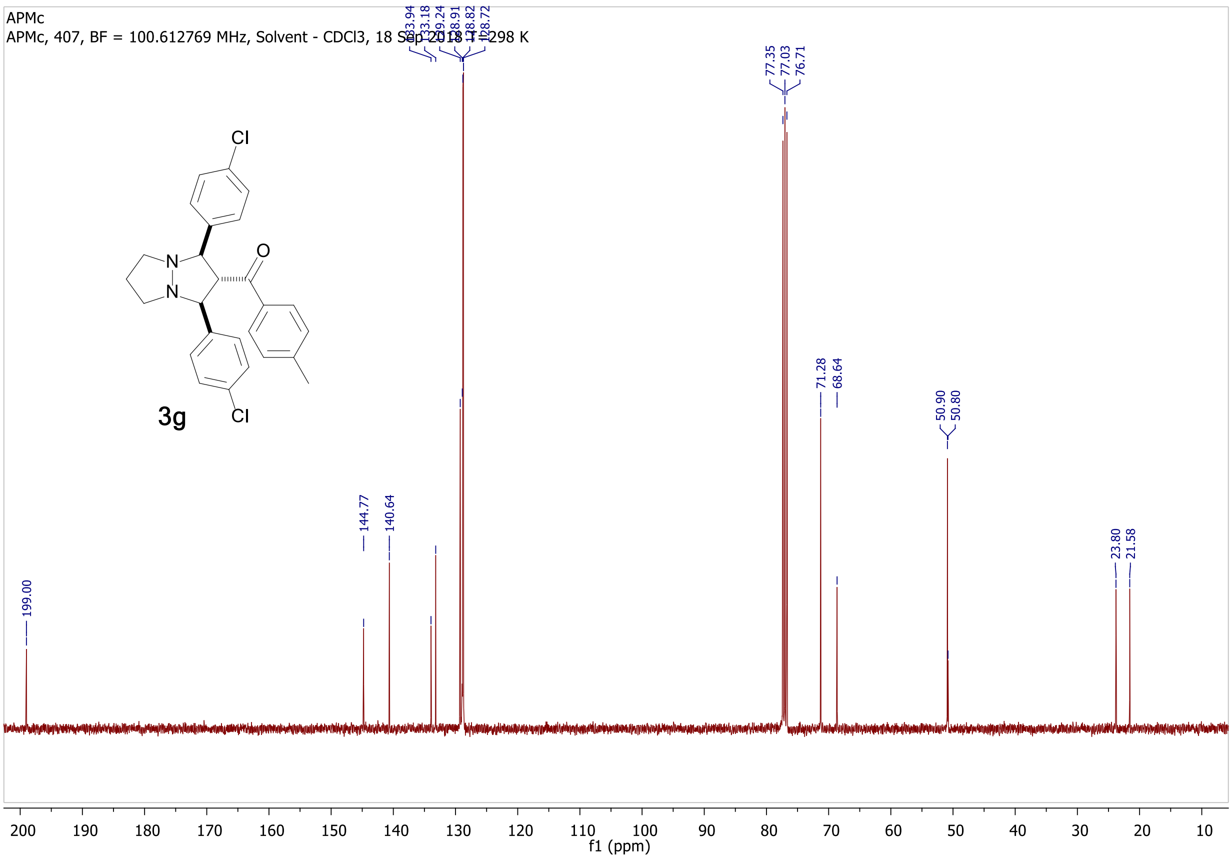
APMc
APMc, 407, BF = 100.612769 MHz, Solvent - CDCl3, 18 Sep 2018 T=298 K
3g
f1 (ppm)

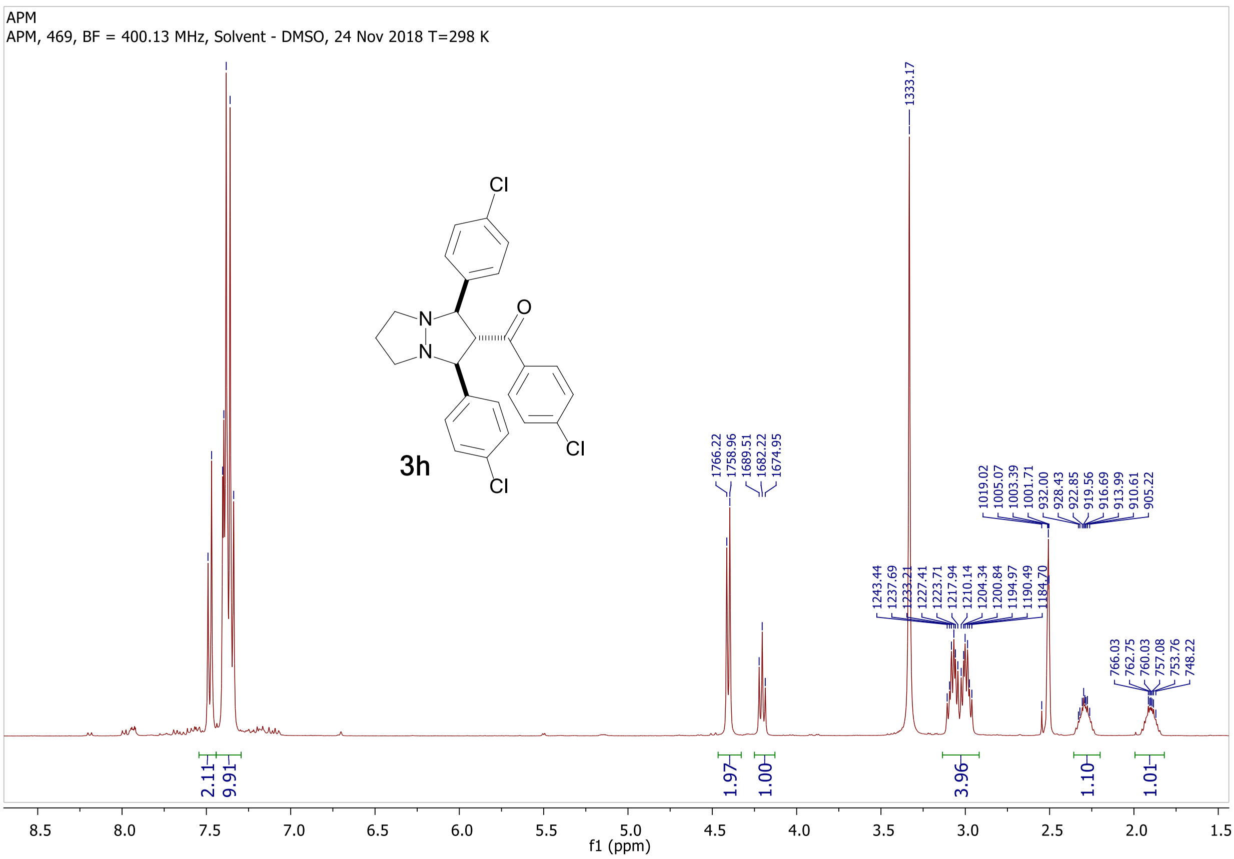

APM
APM, 469, BF = 400.13 MHz, Solvent - DMSO, 24 Nov 2018 T=298 K
3h
f1 (ppm)

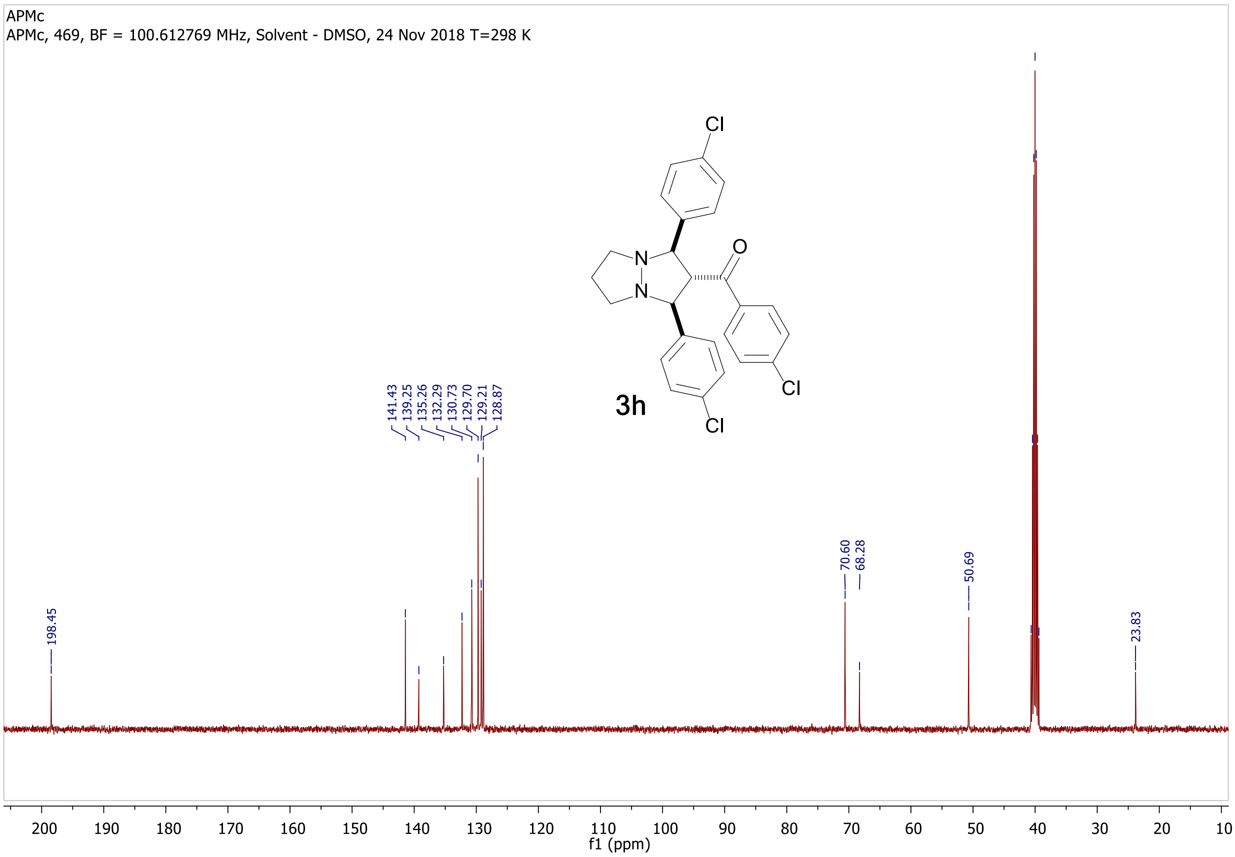

APMc
APMc, 469, BF = 100.612769 MHz, Solvent - DMSO, 24 Nov 2018 T=298 K
3h
f1 (ppm)

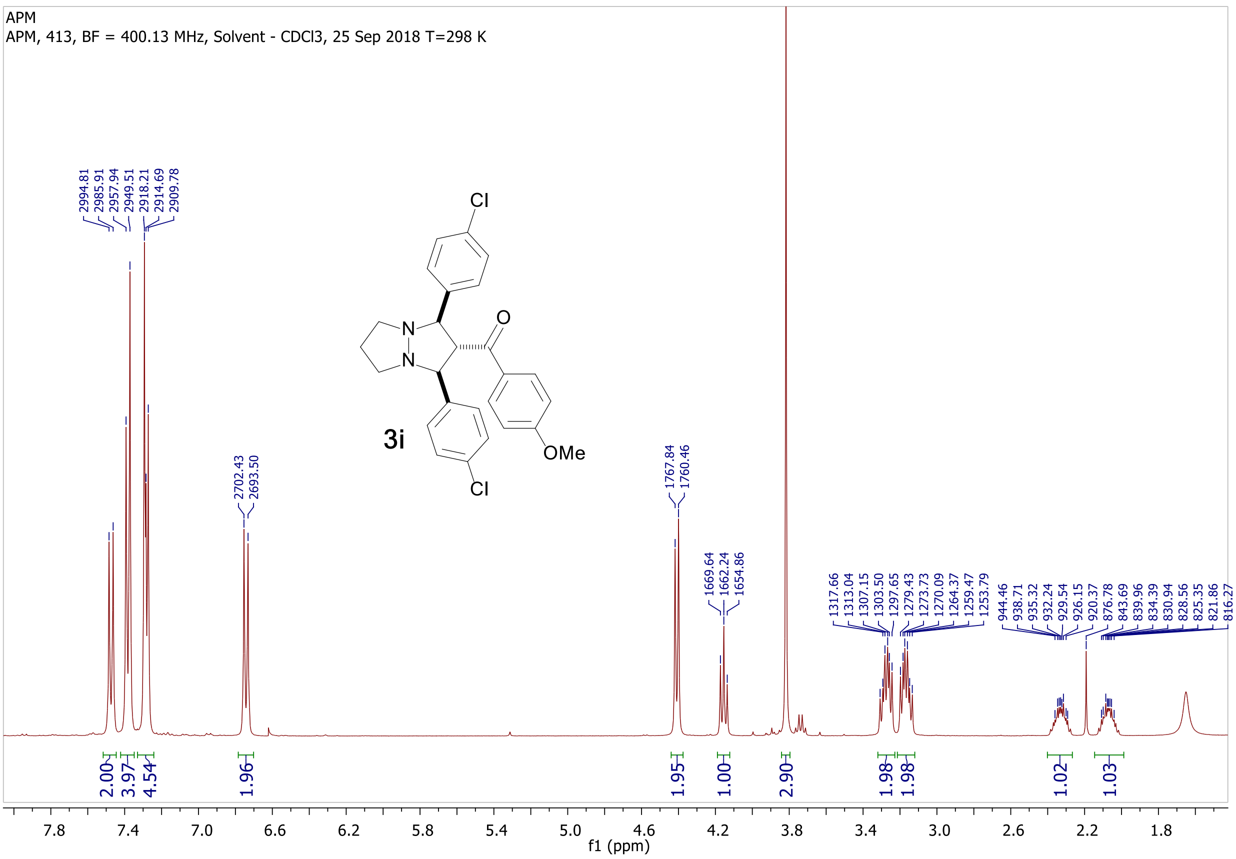
APM
APM, 413, BF = 400.13 MHz, Solvent - CDCl3, 25 Sep 2018 T=298 K
2994.81
2985.91
2957.94
2949.51
2918.21
2914.69
2909.78
2702.43
2693.50
1767.84
1760.46
1669.64
1662.24
1654.86
1317.66
1313.04
1307.15
1303.50
1297.65
1279.43
1273.73
1270.09
1264.37
1259.47
1253.79
944.46
938.71
935.32
932.24
929.54
926.15
920.37
876.78
843.69
839.96
834.39
830.94
828.56
825.35
821.86
816.27
Cl
N
N
O
3i
Cl
OMe
2.00
3.97
4.54
1.96
1.95
1.00
2.90
1.98
1.98
1.02
1.03
7.8
7.4
7.0
6.6
6.2
5.8
5.4
5.0
4.6
4.2
3.8
3.4
3.0
2.6
2.2
1.8
f1 (ppm)

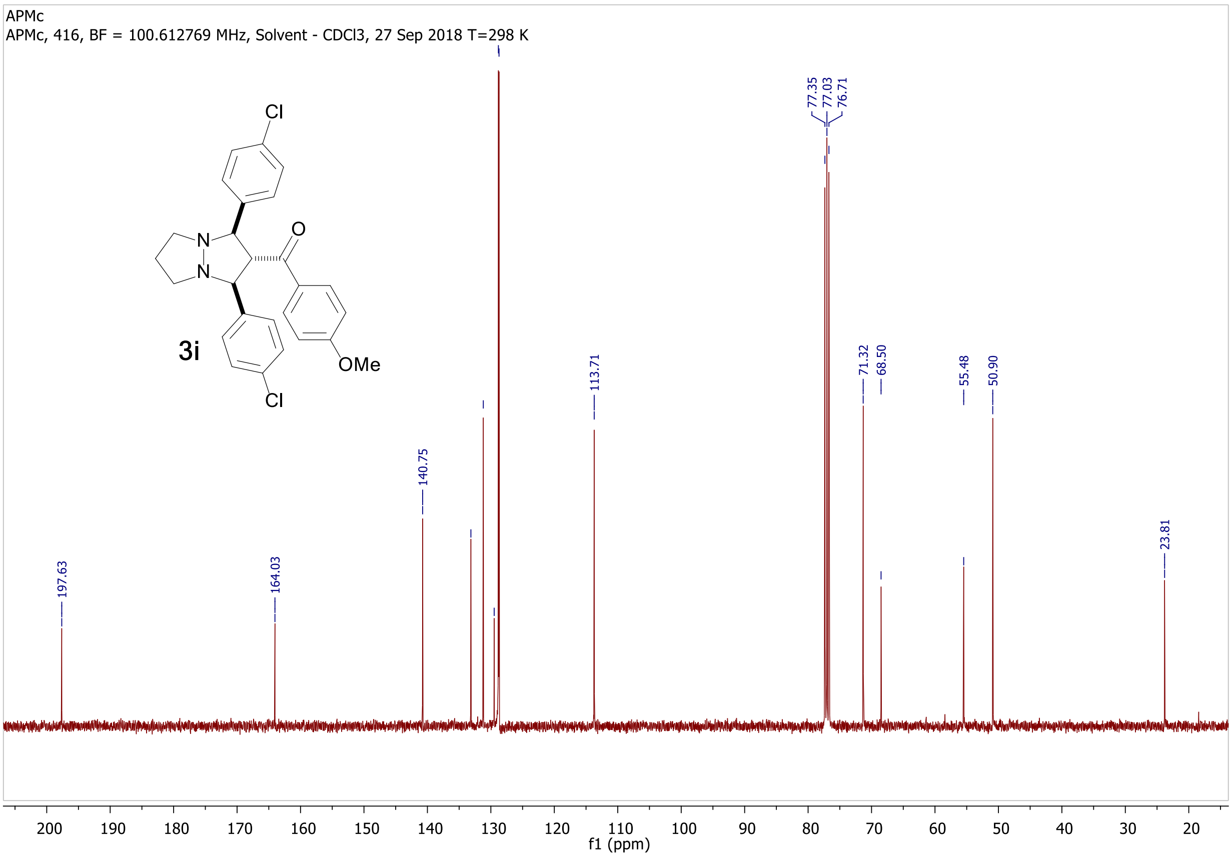
APMc
APMc, 416, BF = 100.612769 MHz, Solvent - CDCl3, 27 Sep 2018 T=298 K
77.35
77.03
76.71
Cl
N
N
O
3i
Cl
OMe
113.71
71.32
68.50
55.48
50.90
140.75
23.81
197.63
164.03
200
190
180
170
160
150
140
130
120
110
100
90
80
70
60
50
40
30
20
f1 (ppm)

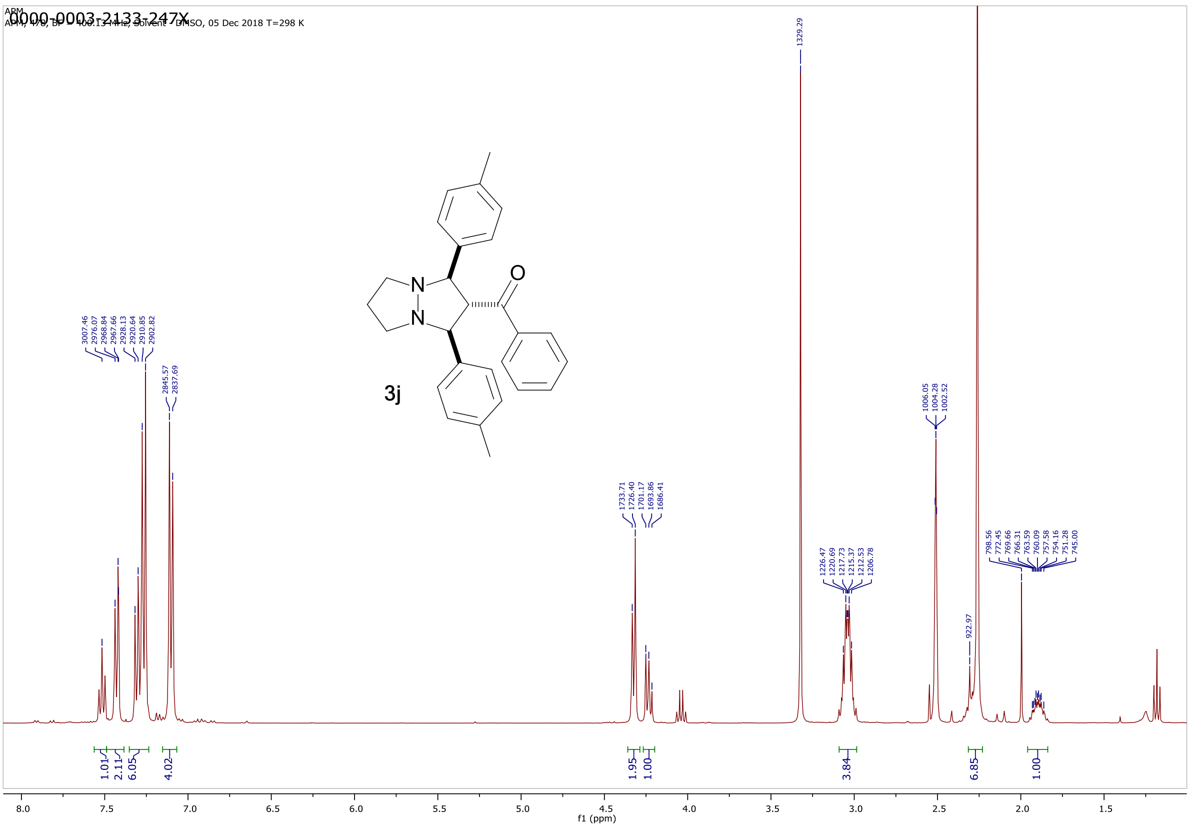
0000-0003-2133-247X
3j
f1 (ppm)

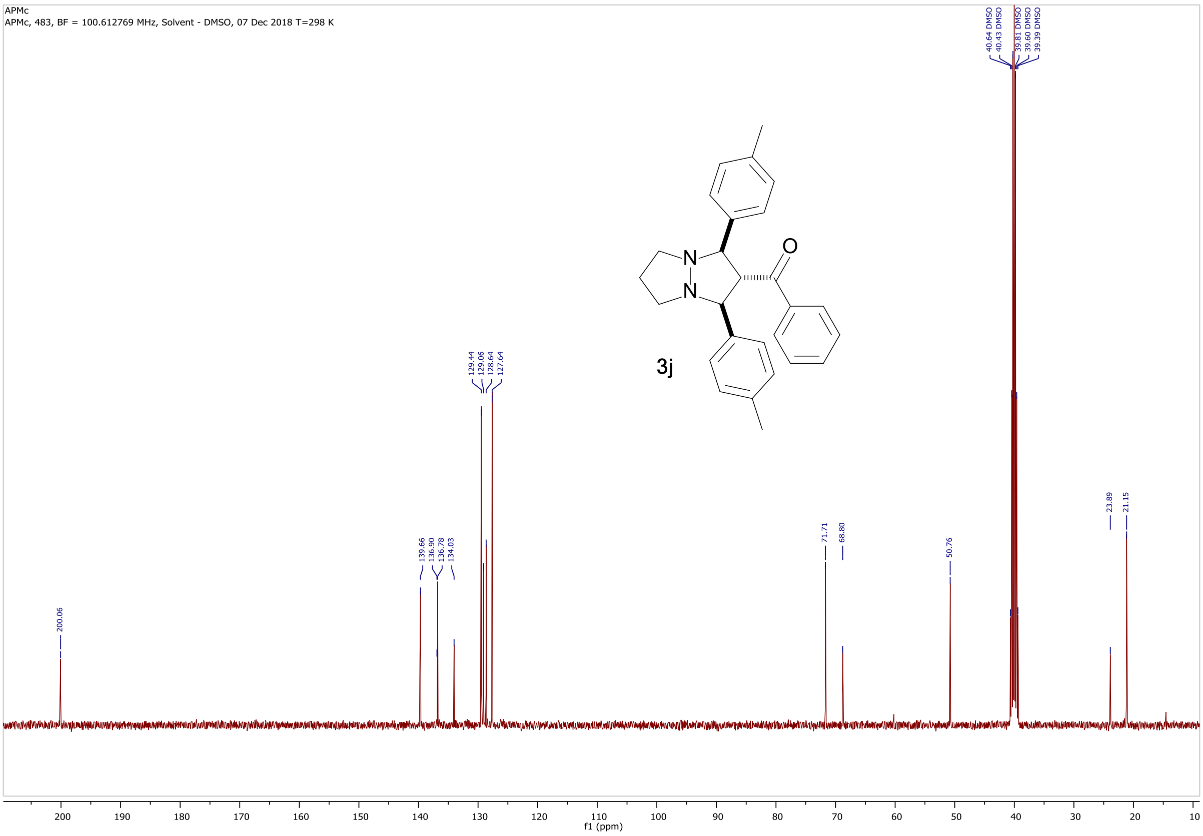
APMc
APMc, 483, BF = 100.612769 MHz, Solvent - DMSO, 07 Dec 2018 T=298 K
3j
f1 (ppm)

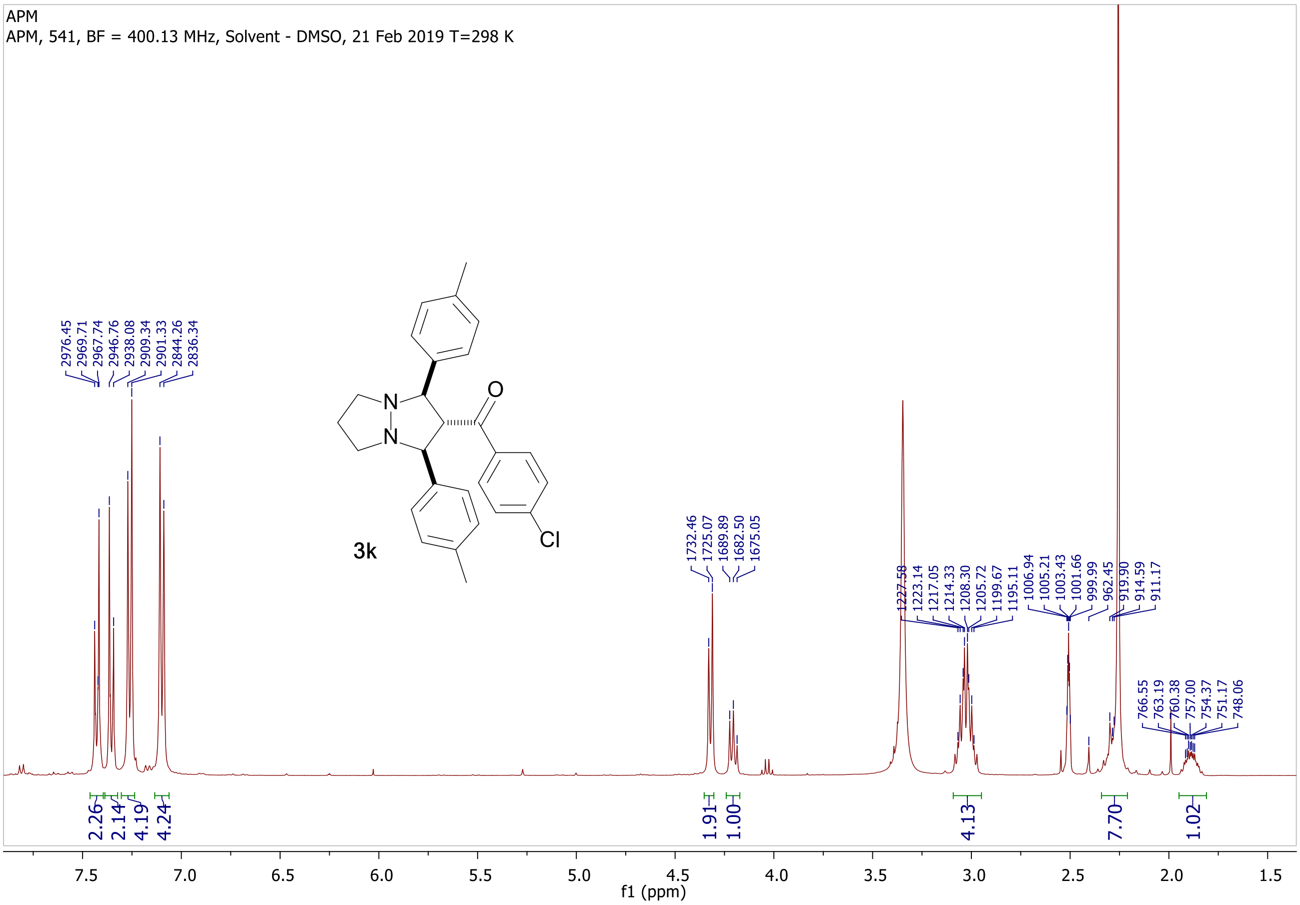
APM
APM, 541, BF = 400.13 MHz, Solvent - DMSO, 21 Feb 2019 T=298 K
2976.45
2969.71
2967.74
2946.76
2938.08
2909.34
2901.33
2844.26
2836.34
1732.46
1725.07
1689.89
1682.50
1675.05
1227.58
1223.14
1217.05
1214.33
1208.30
1205.72
1199.67
1195.11
1006.94
1005.21
1003.43
1001.66
999.99
962.45
919.90
914.59
911.17
766.55
763.19
760.38
757.00
754.37
751.17
748.06
N
N
O
Cl
3k
2.26
2.14
4.19
4.24
1.91
1.00
4.13
7.70
1.02
f1 (ppm)

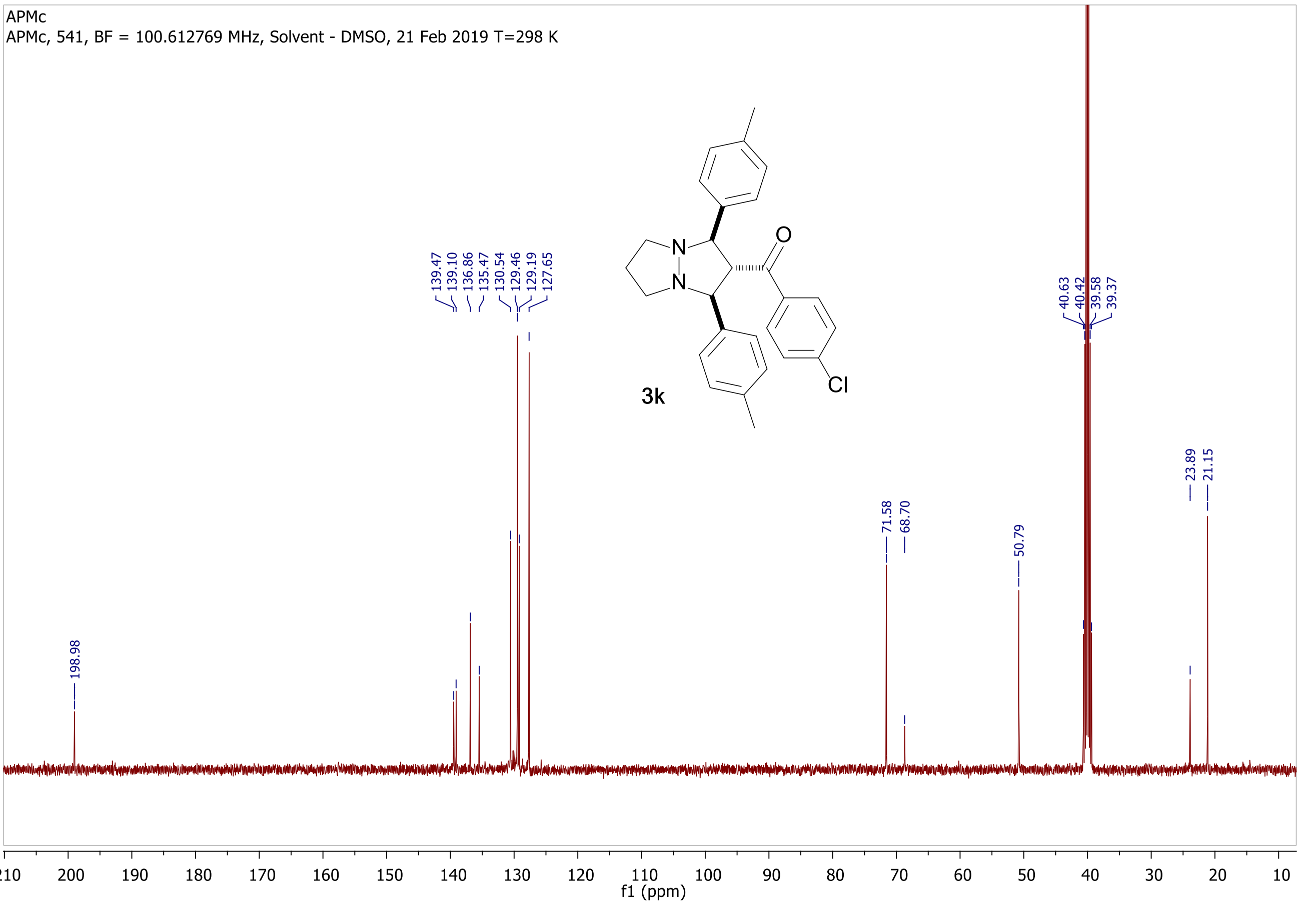
APMc
APMc, 541, BF = 100.612769 MHz, Solvent - DMSO, 21 Feb 2019 T=298 K
198.98
139.47
139.10
136.86
135.47
130.54
129.46
129.19
127.65
71.58
68.70
50.79
40.63
40.42
39.58
39.37
23.89
21.15
N
N
O
Cl
3k
f1 (ppm)

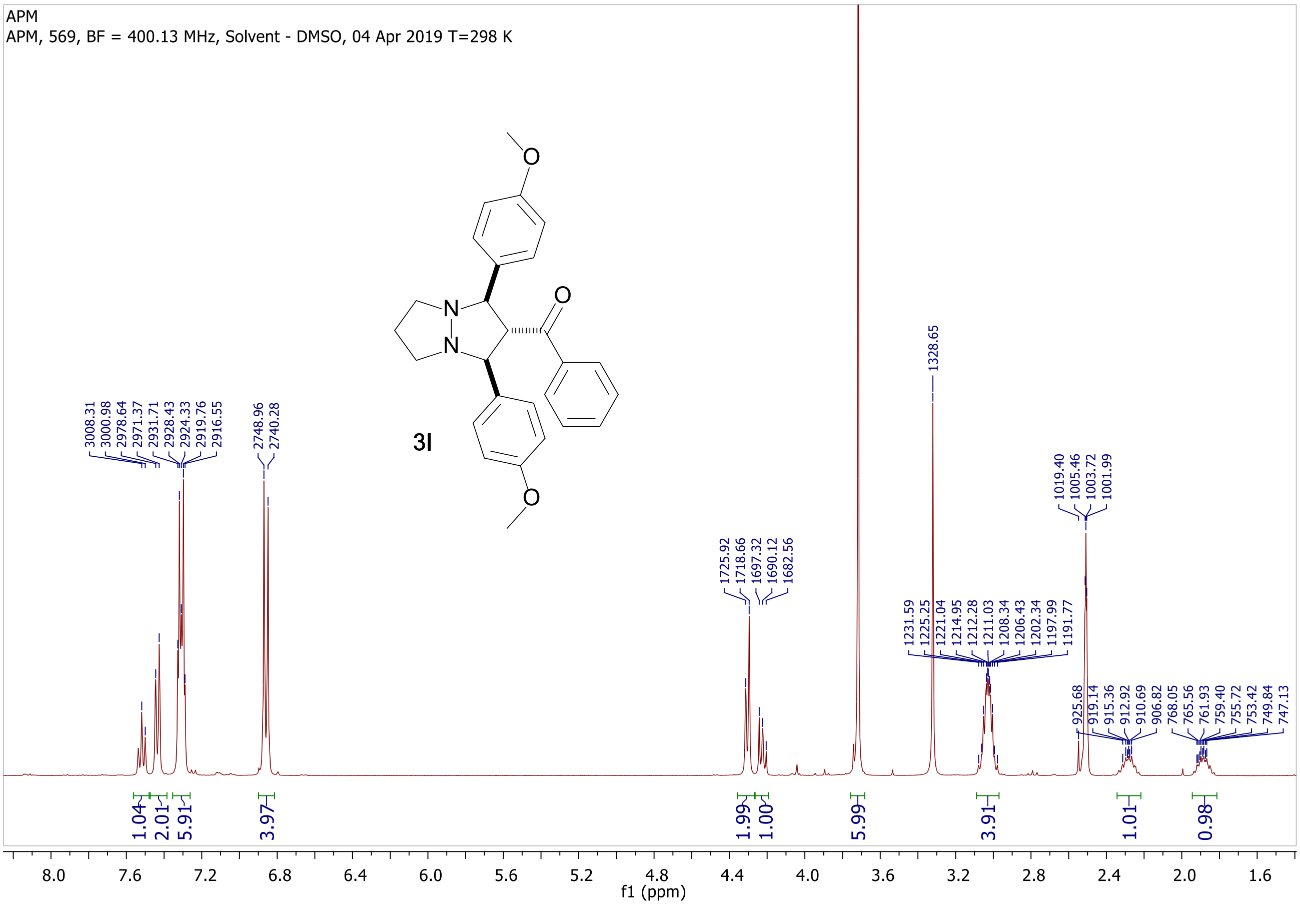
APM
APM, 569, BF = 400.13 MHz, Solvent - DMSO, 04 Apr 2019 T=298 K
3l
f1 (ppm)

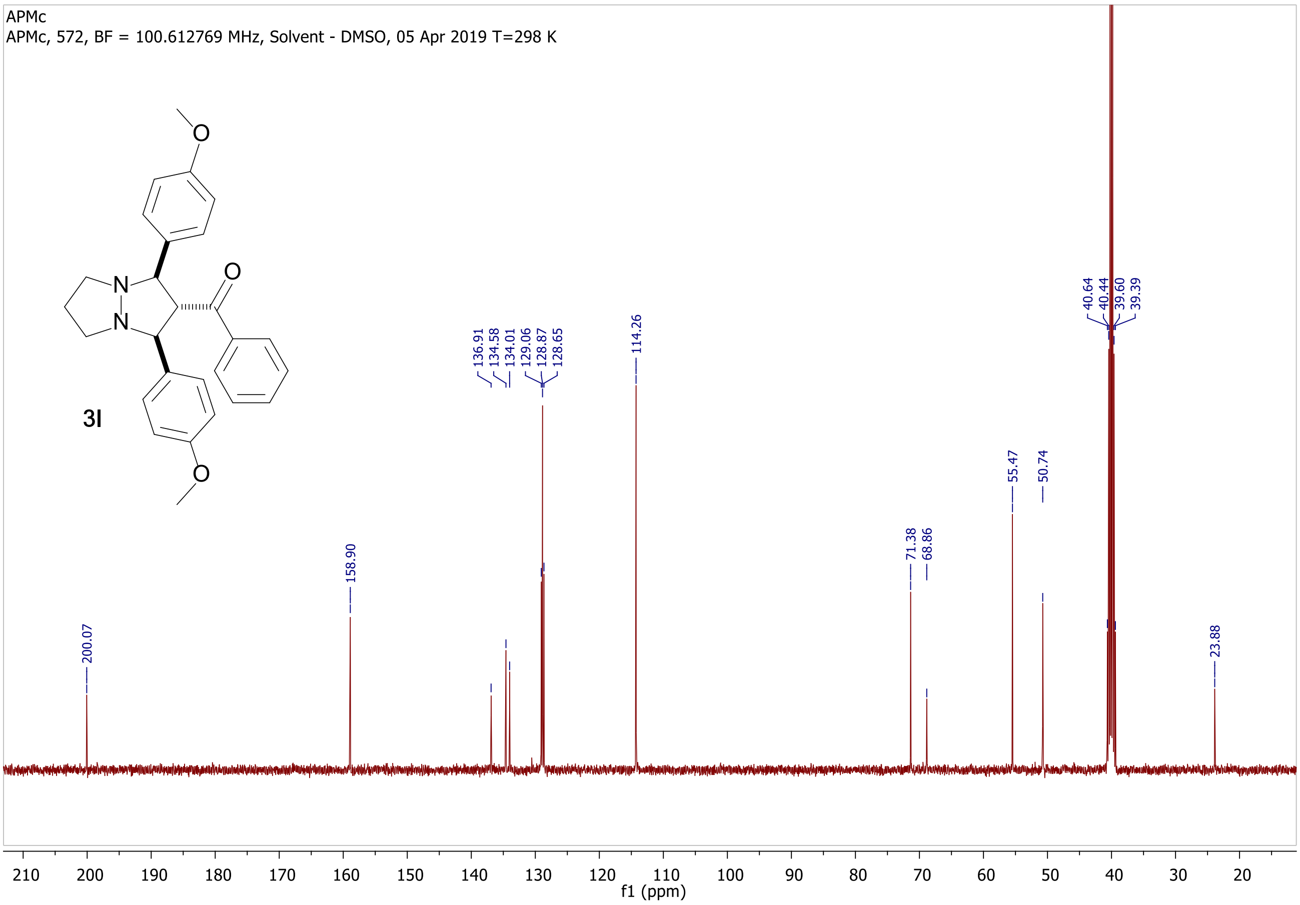
APMc
APMc, 572, BF = 100.612769 MHz, Solvent - DMSO, 05 Apr 2019 T=298 K
3l
200.07
158.90
136.91
134.58
134.01
129.06
128.87
128.65
114.26
71.38
68.86
55.47
50.74
40.64
40.44
39.60
39.39
23.88
f1 (ppm)

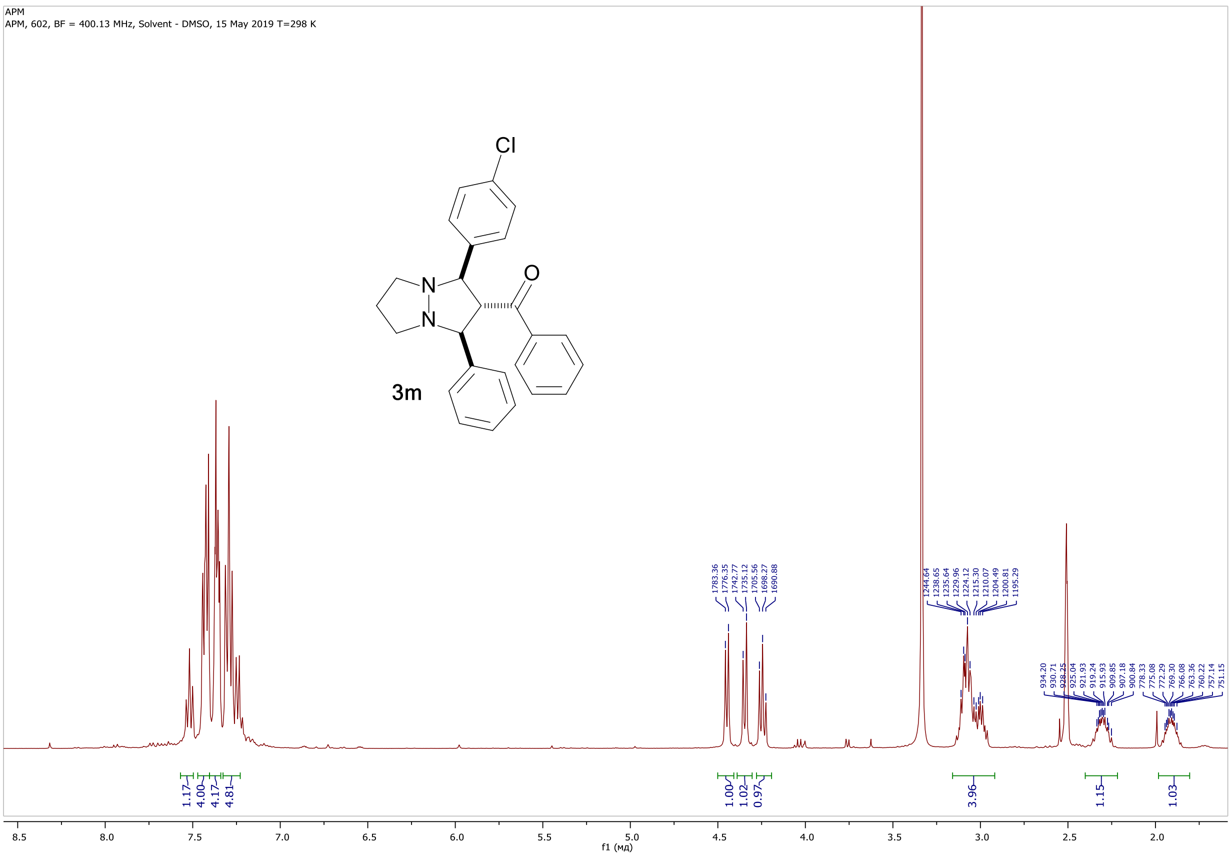
APM
APM, 602, BF = 400.13 MHz, Solvent - DMSO, 15 May 2019 T=298 K
Cl
N
N
O
3m
f1 (мд)

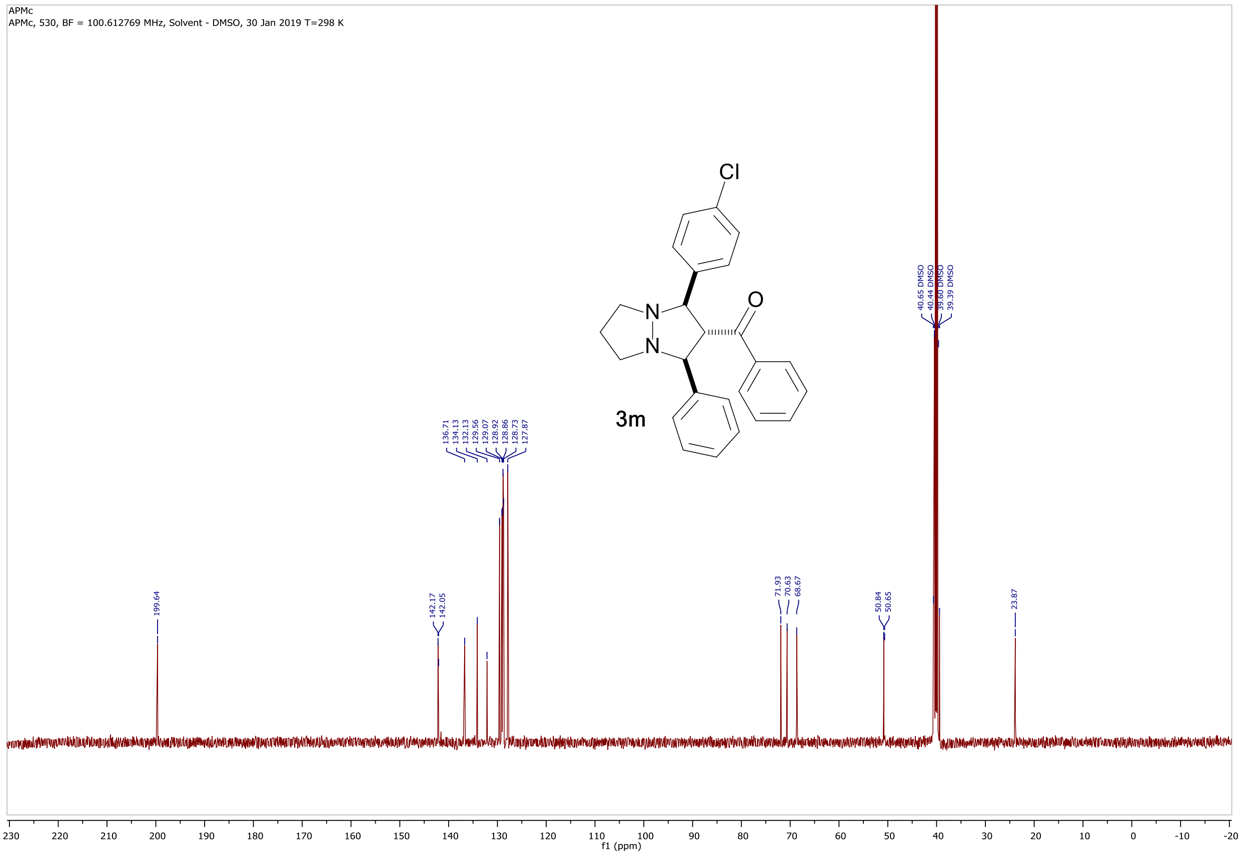
APMc
APMc, 530, BF = 100.612769 MHz, Solvent - DMSO, 30 Jan 2019 T=298 K
Cl
N
N
O
3m
f1 (ppm)

APM
APM, 628, BF = 400.13 MHz, Solvent - CDCl3, 17 Jun 2019 T=298 K

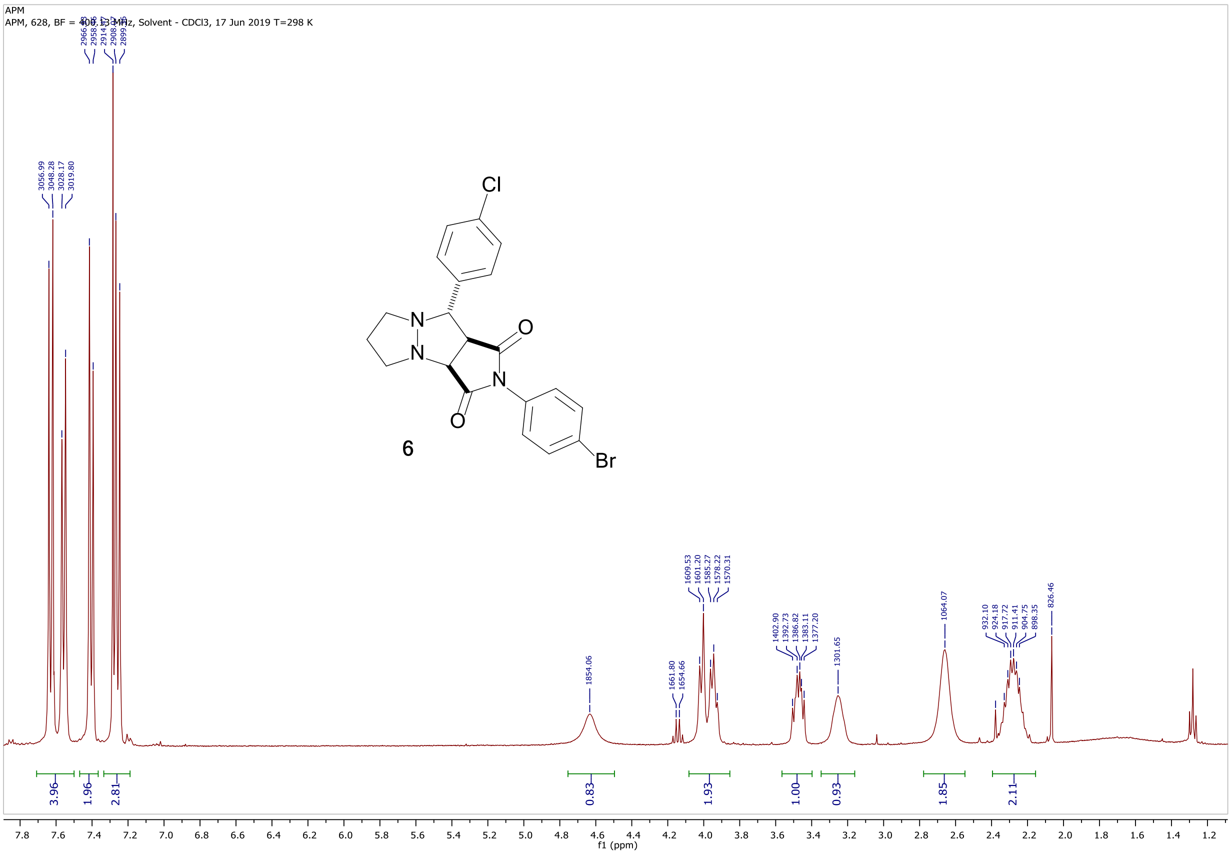

APMc
APMc, 637, BF = 100.612769 MHz, Solvent - DMSO, 26 Jun 2019 T=298 K

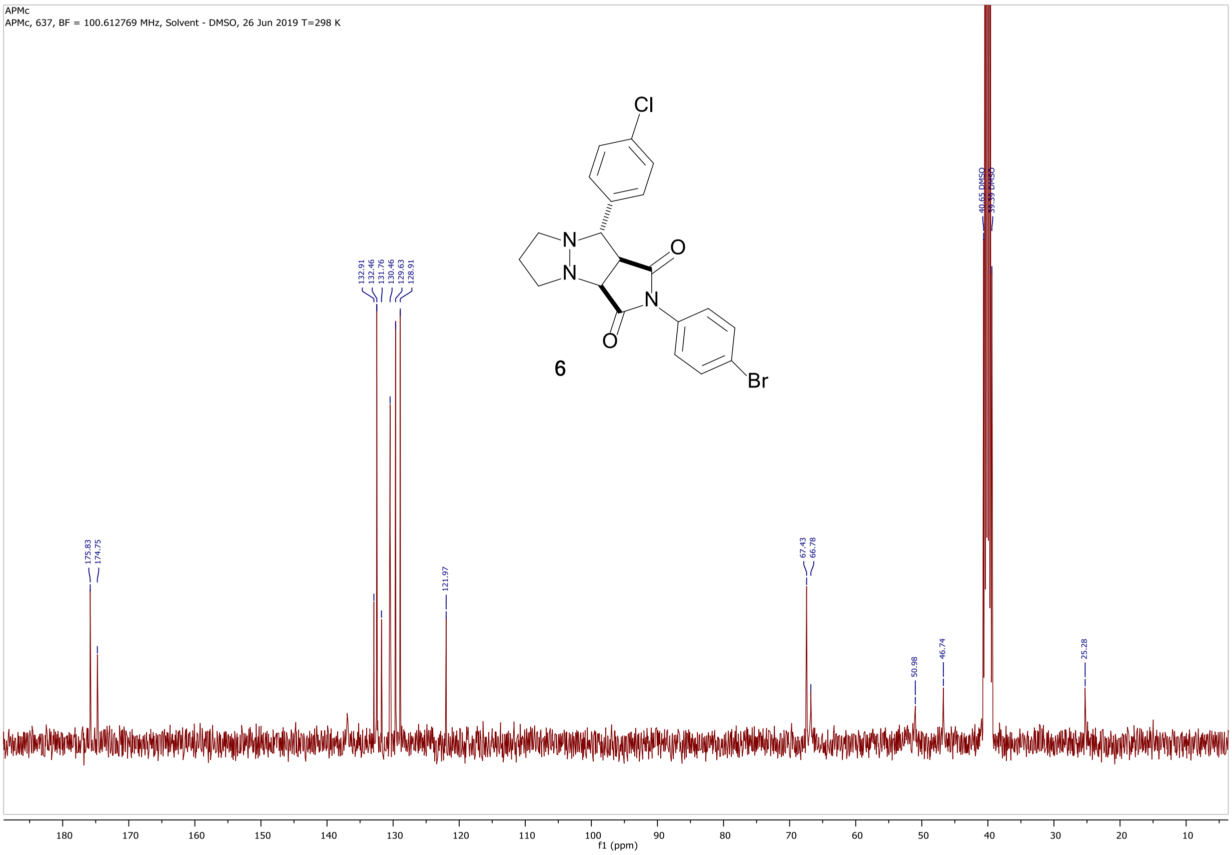

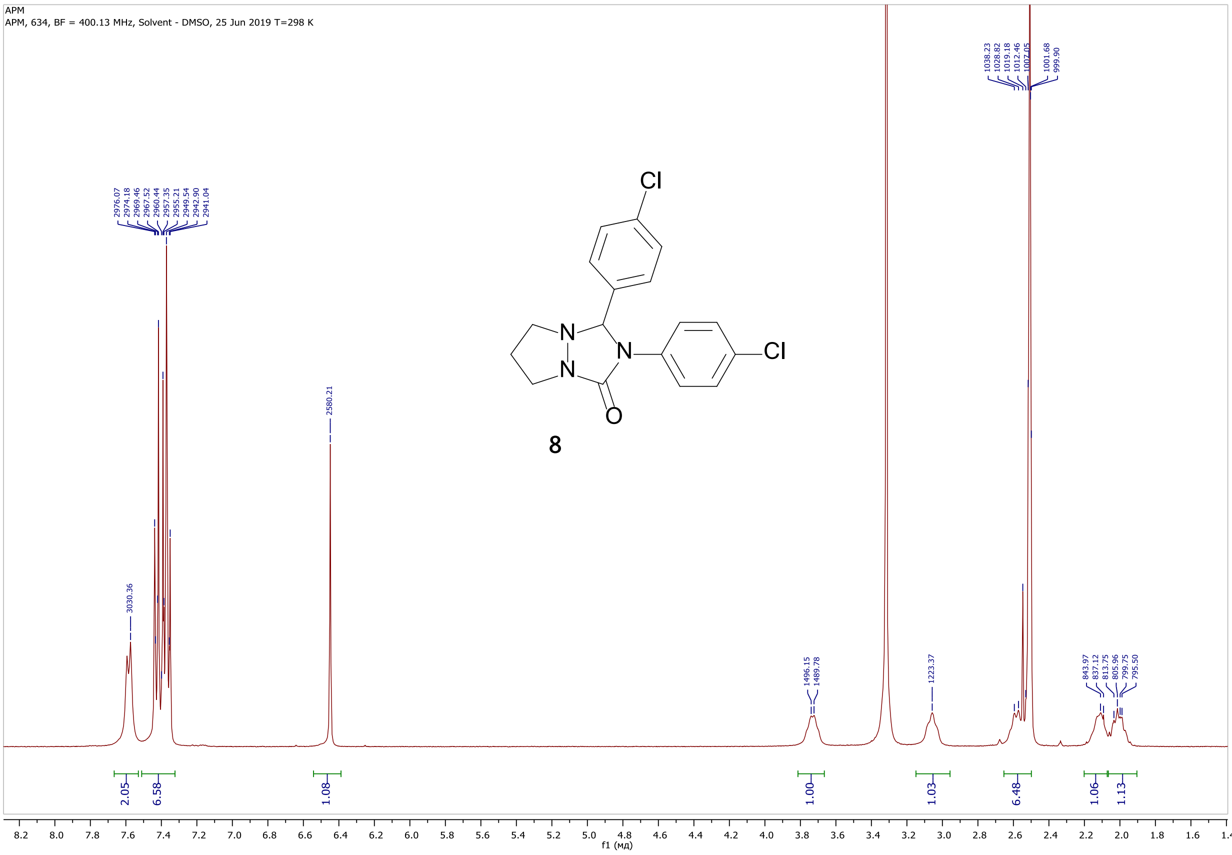
APM
APM, 634, BF = 400.13 MHz, Solvent - DMSO, 25 Jun 2019 T=298 K
Cl
N
N
N
Cl
O
8
2.05
6.58
1.08
1.00
1.03
6.48
1.06
1.13
f1 (мд)

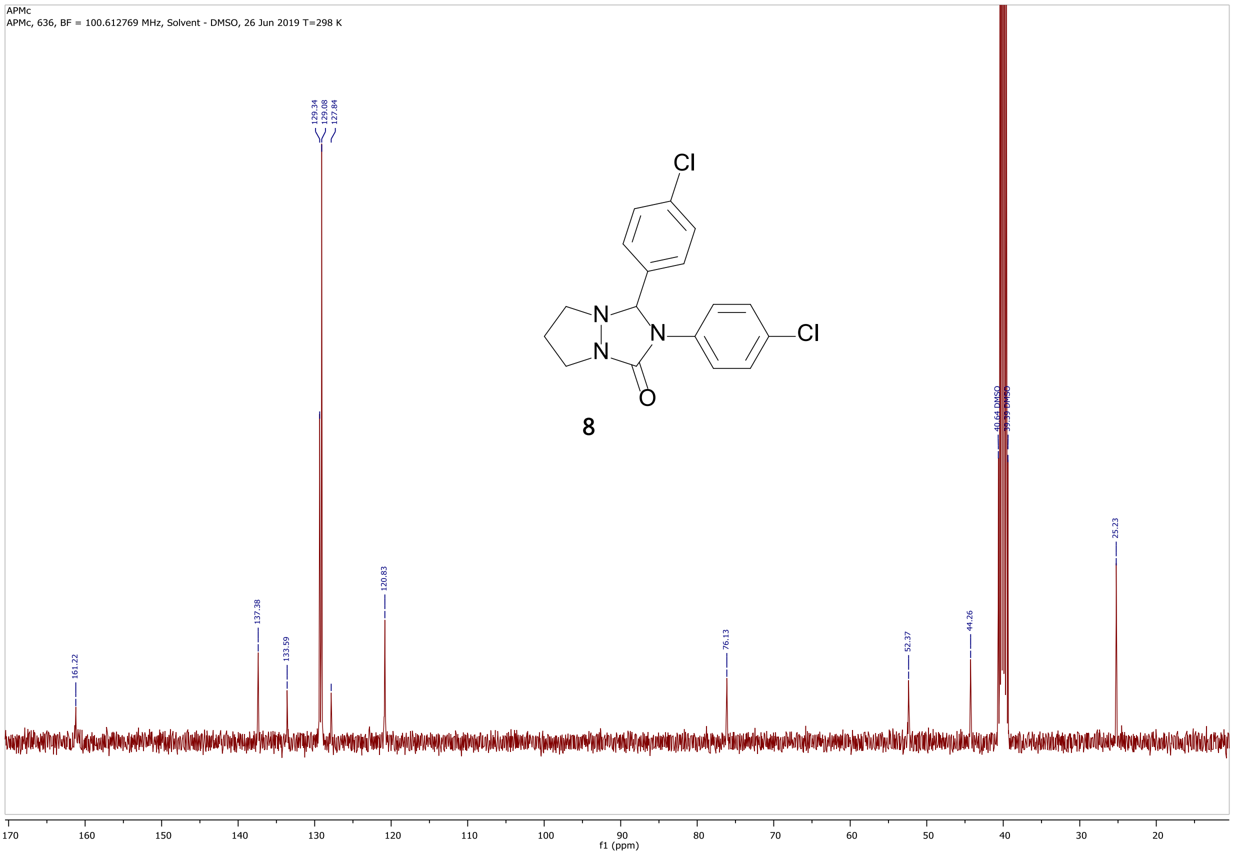
APMc
APMc, 636, BF = 100.612769 MHz, Solvent - DMSO, 26 Jun 2019 T=298 K
Cl
N
N
N
Cl
O
8
f1 (ppm)

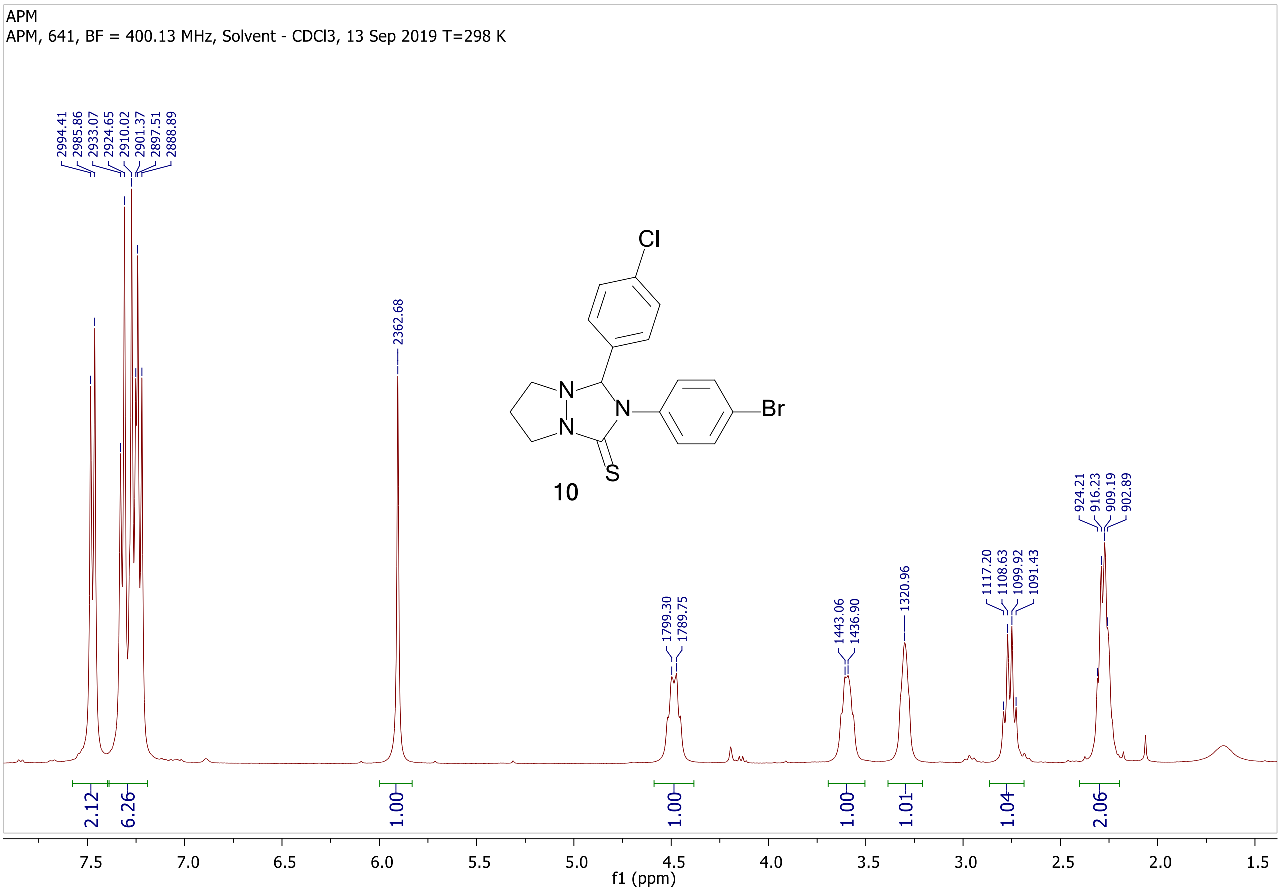
APM
APM, 641, BF = 400.13 MHz, Solvent - CDCl3, 13 Sep 2019 T=298 K
2994.41
2985.86
2933.07
2924.65
2910.02
2901.37
2897.51
2888.89
2362.68
1799.30
1789.75
1443.06
1436.90
1320.96
1117.20
1108.63
1099.92
1091.43
924.21
916.23
909.19
902.89
Cl
N
N
N
Br
S
10
2.12
6.26
1.00
1.00
1.00
1.01
1.04
2.06
7.5
7.0
6.5
6.0
5.5
5.0
4.5
4.0
3.5
3.0
2.5
2.0
1.5
f1 (ppm)

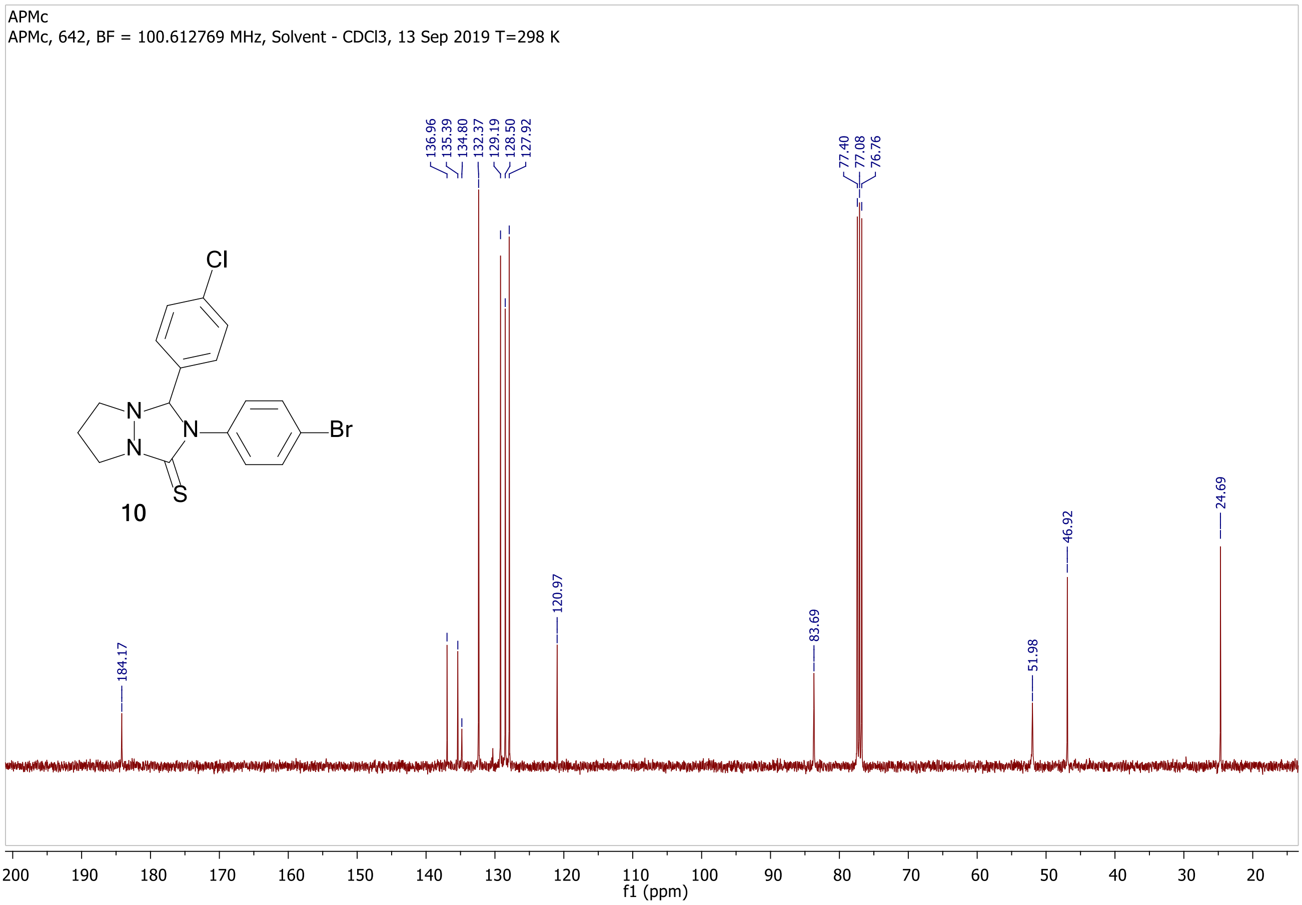
APMc
APMc, 642, BF = 100.612769 MHz, Solvent - CDCl3, 13 Sep 2019 T=298 K
136.96
135.39
134.80
132.37
129.19
128.50
127.92
77.40
77.08
76.76
Cl
N
N
N
Br
S
10
184.17
120.97
83.69
51.98
46.92
24.69
200
190
180
170
160
150
140
130
120
110
100
90
80
70
60
50
40
30
20
f1 (ppm)
